# A Phase 3, Randomized Trial Investigating the Safety, Tolerability, and Immunogenicity of V116, an Adult-Specific Pneumococcal Conjugate Vaccine, in Pneumococcal Vaccine-Naïve Adults 18–64 Years of Age at Increased Risk of Pneumococcal Disease, STRIDE-8

**DOI:** 10.1093/cid/ciaf604

**Published:** 2025-11-10

**Authors:** Paul T Scott, Jayani Pathirana, Akira Kato, Richard Tytus, Carlos M Perez, Nigel Leslie Gilchrist, Hidemi Kanou, Kwang Ha Yoo, Grzegorz Kania, Michael Nissen, Amy Falk Russell, Doreen Fernsler, Muhammad Waleed, Jianing Li, Ulrike K Buchwald, Heather L Platt

**Affiliations:** Global Clinical Development, Merck & Co., Inc., Rahway, New Jersey, USA; Global Clinical Development, Merck & Co., Inc., Rahway, New Jersey, USA; Department of Gastroenterology and Hepatology, Japan Community Health Care Organization Shimonoseki Medical Center, Shimonoseki, Yamaguchi, Japan; Faculty of Family Medicine, McMaster University, Hamilton, Ontario, Canada; Faculty of Medicine, San Sebastian University, Santiago, Chile; CGM Research Trust, Christchurch, New Zealand; Medical Corporation Applied Bio-Pharmatech Kurume Clinical Pharmacology Clinic, Kurume-shi, Fukuoka, Japan; Department of Internal Medicine, Konkuk University Medical Center, Seoul, Republic of Korea; Faculty of Health Sciences, Medical University of Lublin, Lublin, Poland; Queensland Adult Specialist Immunisation Service, The Prince Charles Hospital, Chermside, QLD, Australia; Infectious Diseases Unit, Royal Brisbane and Women's Hospital, Herston, QLD, Australia; Global Clinical Development, Merck & Co., Inc., Rahway, New Jersey, USA; Global Clinical Development, Merck & Co., Inc., Rahway, New Jersey, USA; Global Clinical Development, Merck & Co., Inc., Rahway, New Jersey, USA; Global Clinical Development, Merck & Co., Inc., Rahway, New Jersey, USA; Global Clinical Development, Merck & Co., Inc., Rahway, New Jersey, USA; Global Clinical Development, Merck & Co., Inc., Rahway, New Jersey, USA

**Keywords:** adults, at-risk, chronic disease, pneumococcal disease, pneumococcal conjugate vaccine

## Abstract

**Background:**

Pneumococcal disease (PD) is a major cause of hospitalization and mortality in adults. Individuals with certain chronic illnesses are at increased risk for PD.

**Methods:**

This phase 3, randomized, double-blind, active comparator-controlled trial (NCT05696080) evaluated the safety and immunogenicity of 21-valent pneumococcal conjugate vaccine (V116) in adults 18–64 years of age with ≥1 condition associated with increased risk of PD (diabetes mellitus or kidney, heart, liver, or lung disease). Participants were given a single dose of either V116 followed by placebo or 15-valent pneumococcal conjugate vaccine (PCV15), followed by 23-valent pneumococcal polysaccharide vaccine (PPSV23) 8 weeks later. Immune responses were evaluated by opsonophagocytic activity (OPA) geometric mean titers (GMTs) and immunoglobulin G (IgG) geometric mean concentrations (GMCs) at 30 days postvaccination (day 30 for V116; week 12 for PCV15 + PPSV23). Proportions of participants who experienced adverse events (AEs) within 5 days postvaccination and serious AEs (SAEs) or deaths during the study were assessed.

**Results:**

V116 was immunogenic for all 21 serotypes contained in the vaccine. OPA GMTs and IgG GMCs following V116 vaccination were comparable to PCV15 + PPSV23 for the 13 serotypes common between vaccine groups. For the eight serotypes unique to V116, immune responses were higher following V116 compared with PCV15 + PPSV23. V116 was well tolerated compared with PCV15 + PPSV23; no vaccine-related SAEs or deaths were reported.

**Conclusions:**

V116 elicits robust immune responses and is well tolerated in adults 18–64 years of age with conditions associated with an increased risk of PD.

## Teaser

This article describes results of a phase 3 study assessing the safety, tolerability, and immunogenicity of 21-valent pneumococcal conjugate vaccine, V116, in adults at increased risk for pneumococcal disease. V116 was well tolerated and elicited robust immune responses.

*Streptococcus pneumoniae* is the causative agent of pneumococcal disease (PD), which is associated with substantial morbidity and mortality in both children and adults [1–3]. Certain chronic medical conditions, such as diabetes and heart, kidney, liver, and lung disease, increase individual risk of PD [4–6]. Individuals with chronic illnesses have been reported to have up to 10.0- and 6.2-times greater incidence rates of invasive PD (IPD) and pneumococcal pneumonia, respectively, compared with healthy individuals [4–6]. The presence of chronic conditions also substantially increases the risk of PD-related hospitalization and death [4, 5].

The use of pneumococcal conjugate vaccines (PCVs) in infants and children has decreased the overall incidence of PD, including IPD, in children <5 years of age [7, 8]. Although there is evidence of indirect protection of adults from infant vaccination programs, there is a substantial remaining burden of PD in adult populations [7, 8]. Adults at increased risk of PD may especially benefit from vaccination that protects against IPD and pneumonia-related mortality [9].

V116 (CAPVAXIVE^TM^, Merck Sharp & Dohme LLC, a subsidiary of Merck & Co., Inc., Rahway, NJ, USA [MSD]) is a 21-valent, adult-specific PCV designed to include serotypes associated with IPD in adults living in regions with well-established pediatric pneumococcal vaccination programs. V116 is approved in adults ≥18 years of age for the prevention of IPD and pneumonia in the United States and the European Union, for the prevention of IPD in Canada, and for the prevention of PD in Australia [10–13]. The serotypes in V116 are responsible for 81% and 85% of residual IPD in at-risk adults 19–64 years of age and ≥65 years of age in the United States, respectively [14]. V116 contains eight unique serotypes (excluding cross-reactive serotypes) that are not included in any other currently licensed PCV, and these serotypes accounted for ∼20% and ∼30% of IPD cases in the United States from 2018 to 2022 among at-risk adults 19–64 years of age and ≥65 years of age, respectively [14].

In the United States, options for pneumococcal vaccination of adults at increased risk of PD due to underlying conditions are a sequential regimen of 15-valent PCV (PCV15; VAXNEUVANCE™, MSD) [15, 16] plus 23-valent pneumococcal polysaccharide vaccine (PPSV23; Pneumovax^Ⓡ^23, MSD) [17, 18], 20-valent PCV (PCV20; PREVNAR 20^Ⓡ^, Pfizer) [19], or V116 [20, 21]. At-risk adults may benefit from V116 vaccination, as it provides broader protection against PD compared with any other currently licensed PCV. This article reports findings from a randomized, global, phase 3 study that evaluated the safety, tolerability, and immunogenicity of V116 in pneumococcal vaccine-naïve adults 18–64 years of age at increased risk of PD due to diabetes mellitus or chronic heart, kidney, liver, or lung disease.

## METHODS

### Study Design and Participants

STRIDE-8 (Protocol #V116-008, NCT05696080) was an international, phase 3, randomized, double-blind, active comparator-controlled trial of V116 in pneumococcal vaccine-naïve adults 18–64 years of age at increased risk of PD. The first participant first visit was 13 February 2023, and the last participant last visit was 16 February 2024; the last data available date was 1 March 2024. The trial included study sites in Australia, Canada, Chile, Japan, New Zealand, Poland, South Korea, and the United States. A full list of study investigators can be found in Supplementary Table 1.

Participants had at least one of the following chronic stable underlying medical conditions considered to increase the risk of PD: diabetes mellitus (receiving treatment with ≥1 approved antidiabetic medication, with all glycated hemoglobin [HbA_1c_] measurements ≤9% within 6 months prior to vaccination); chronic heart disease (including cardiomyopathy, congestive heart failure, or noncyanotic congenital heart disease); confirmed diagnosis of chronic kidney disease with >3 months duration (with glomerular filtration rate [GFR] 45–89 mL/min/1.73 m^2^ with albuminuria <3 mg/mmol, or GFR 45–≥90 mL/min/1.73 m^2^ with albuminuria 3–30 mg/mmol); compensated chronic liver disease; or chronic lung disease (including chronic obstructive pulmonary disease or mild/moderate persistent asthma). Participants’ chronic medical conditions were assessed as stable by an investigator. Participants also had ≤1 hospitalization directly related to their risk condition within 3 months before first study vaccination. A detailed list of inclusion criteria for underlying medical conditions can be found in Supplementary Table 2.

Exclusion criteria included the presence of underlying medical conditions such as: severe pulmonary hypertension or Eisenmenger syndrome; autoimmune-related kidney disease, chronic kidney failure, a reversible cause of kidney disease, nephrotic syndrome, or any ineligible Kidney Disease Improving Global Outcomes-recommended stage of GFR and albuminuria; history of active hepatitis within 3 months prior to the first study vaccination; diabetic ketoacidosis or symptomatic hypoglycemia (≥2 severe episodes) within 3 months before receiving the first study vaccination; or occurrence of certain cardiovascular events (including myocardial infarction, acute coronary syndrome, transient ischemic attack, or ischemic or hemorrhagic stroke) within 3 months prior to the first study vaccination. A detailed list of exclusion criteria for underlying medical conditions can be found in Supplementary Table 2. In addition, participants were excluded for prior or concomitant receipt of any pneumococcal vaccine, with the exception of receipt in early childhood (prior to 5 years of age).

The study was conducted in accordance with principles of Good Clinical Practice and was approved by the appropriate institutional review boards and regulatory agencies. Informed consent was obtained from all individuals involved in the study.

### Randomization and Masking

**Figure 1.**
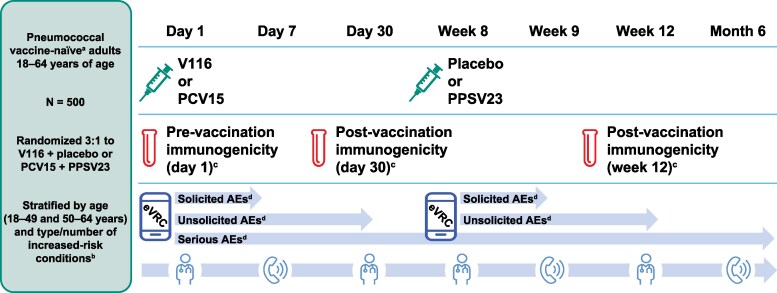
Study design. Abbreviations: AE, adverse event; eVRC, electronic vaccination report card; PCV15, 15-valent pneumococcal conjugate vaccine; PPSV23, 23-valent pneumococcal polysaccharide vaccine; V116, 21-valent adult-specific pneumococcal conjugate vaccine. ^a^Participants who received childhood pneumococcal vaccination before 5 y of age were permitted. ^b^Randomization categories for risk condition stratification were diabetes mellitus only, chronic heart disease only, chronic kidney disease only, chronic liver disease only, chronic lung disease only, and ≥2 increased-risk conditions. ^c^The per-protocol population comprised all randomized participants without deviations from the protocol that may substantially affect the results of the immunogenicity endpoints. ^d^The safety population included all randomized participants who received at least one dose of study vaccine. Reported AEs included nonserious AEs occurring within 30 d of vaccination and SAEs occurring from d 1 until the end of study participation.

Participants were randomized in a 3:1 ratio to receive either V116 on day 1 followed by placebo at week 8 (V116 + placebo group), or PCV15 on day 1 followed by PPSV23 at week 8 (PCV15 + PPSV23 group; Figure 1). Randomization was stratified by age (18–49 and 50–64 years of age) and by the type and number of chronic risk conditions. The study aimed to randomize ∼375 participants to the V116 + placebo group and 125 participants to the PCV15 + PPSV23 group, with a 90% evaluability rate assumed for immunogenicity analyses for both groups. See also Supplementary Methods 1.

All personnel involved in the study were blinded to the study intervention assignments. An unblinded pharmacist or qualified study site personnel received, maintained, prepared and/or dispensed, and administered the study interventions. Participant contact with unblinded study site personnel was limited to administration of study vaccines. The clinical biostatistics department of the sponsor generated the randomized allocation schedule for study treatment assignment, which was implemented using an interactive response technology system. See also Supplementary Methods 1.

### Procedures

The vaccines studied in this analyses were V116 [11], PCV15, and PPSV23 [15, 17]. The compositions of these vaccines are included in Supplementary Methods 1.

Vaccines were administered intramuscularly in a single 0.5-mL dose. Blood samples were collected from participants on day 1, day 30, and at week 12 for immunogenicity assays. Functional antibodies were measured using a serotype-specific microcolony multiplexed opsonophagocytic killing assay [22]. Serotype-specific pneumococcal capsular polysaccharide immunoglobulin G (IgG) antibodies were evaluated using a multiplexed electrochemiluminescence [23] assay using custom plates based on Meso Scale Discovery (Rockville, MD, USA). Both assays were developed at MSD and have been validated.

All participants used an electronic vaccination report card (eVRC) to report solicited injection-site adverse events (AEs), solicited systemic AEs, and daily body temperatures from day 1 through day 5 following each vaccination. Unsolicited AEs were recorded from day 1 through day 30 following vaccination, and serious AEs (SAEs) and deaths were recorded throughout the duration of the study. In addition to using the eVRC and on-site assessment on day 1, day 30, week 8, and week 12, participants were reached by telephone on day 7, week 9, and month 6 to assess for any AEs.

To support the periodic review of safety and tolerability data across the adult V116 phase 3 program, an external unblinded statistician provided unblinded interim safety summaries to an independent external data monitoring committee for their review. The data monitoring committee reviewed interim study results, considered the overall risk and benefit to study participants, and recommended to the executive oversight committee whether the study should continue in accordance with the protocol. See also Supplementary Methods 1.

### Objectives and Analyses

The primary immunogenicity objective was to evaluate the serotype-specific opsonophagocytic activity (OPA) geometric mean titers (GMTs) 30 days following vaccination with V116 (day 30) for the V116 + placebo group and 30 days following vaccination with PPSV23 (week 12) for the PCV15 + PPSV23 group. Serotype-specific OPA responses were assessed for the 13 serotypes common to V116 and PCV15 + PPSV23, as well as for the eight serotypes unique to V116.

Secondary immunogenicity endpoints included serotype-specific IgG geometric mean concentrations (GMCs) postvaccination, as well as serotype-specific geometric mean fold rises (GMFRs) and the proportions of participants with a ≥ 4-fold rise from baseline (day 1) to postvaccination (30 days following vaccination with V116 [day 30] for the V116 + placebo group; 30 days following vaccination with the final dose in the regimen [week 12] for the PCV15 + PPSV23 group) within each vaccination group separately. These endpoints were measured for the 13 serotypes common between V116 and PCV15 + PPSV23, as well as the eight serotypes unique to V116. Immunogenicity analyses were based on the per-protocol population, which was defined as all randomized participants without protocol deviations that could have substantially affected the results of the immunogenicity analyses.

Subgroup analyses were performed by number of baseline increased-risk conditions for PD for serotype-specific OPA GMTs 30 days postvaccination with V116 or PPSV23 within each intervention group.

The primary safety objective was to assess the proportion of participants with AEs following vaccination with V116. The proportions of participants with solicited injection-site AEs and solicited systemic AEs were measured from day 1 to day 5 following each vaccination, and the proportions with vaccine-related SAEs and deaths were measured from day 1 through the duration of the study. Safety analyses included all randomized participants who received at least one dose of study intervention.

Baseline characteristics and demographics, such as age, sex, race, and ethnicity, were collected for the V116 + placebo and PCV15 + PPSV23 groups. In addition to increased-risk conditions, medical history ≤5 years prior to first study vaccination (day 1) was collected.

This was a descriptive study in which no hypotheses were tested. All parameters were summarized via descriptive statistics. For select immunogenicity evaluations, within-group 95% confidence intervals (CIs) were calculated for each vaccination group. For the overall safety evaluation, point estimates and within-group 95% CIs were calculated. Analyses were performed with SAS software version 9.4 (SAS Institute Inc., 2023).

## RESULTS

### Participant Disposition and Characteristics

**Figure 2.**
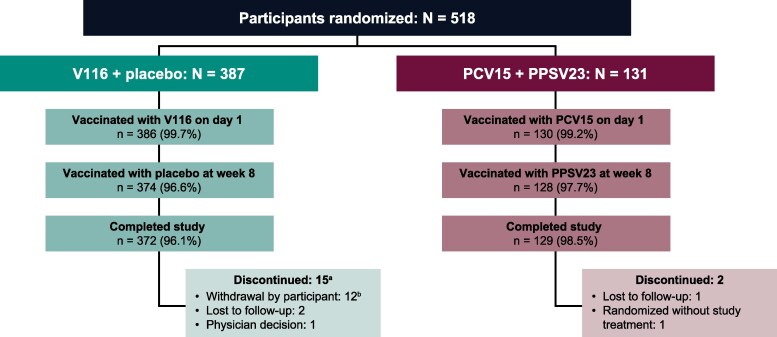
Participant disposition. Abbreviations: *N*, number of participants randomized; *n*, number of participants contributing to analysis; PCV15, 15-valent pneumococcal conjugate vaccine; PPSV23, 23-valent pneumococcal polysaccharide vaccine; V116, 21-valent adult-specific pneumococcal conjugate vaccine. ^a^No trends were observed in the reasons for discontinuation among the participants in the V116 + placebo group. ^b^Reasons for participant withdrawal: logistical difficulties in attending follow-up visits, declined to provide blood samples, declined to receive another injection, and issues related to comorbidities.

A total of 518 participants were randomized, and nearly all participants in the V116 + placebo group (96.1%, *n* = 372) and the PCV15 + PPSV23 group (98.5%, *n* = 129) completed the study (Figure 2).

Baseline characteristics and demographics were evenly distributed across groups (Table 1). The median age was 55 years (range 18–64 years). The majority of participants were White (76.6%), male (54.8%), and not of Hispanic or Latino ethnicity (72.3%). Most (84.1%) participants had a single increased-risk condition for PD. The most common single increased-risk conditions were diabetes mellitus (37.6%), chronic lung disease (19.2%), and chronic heart disease (16.3%). The proportions of participants with specific increased-risk conditions for PD were generally balanced across intervention groups (Table 1).

**Table 1.** Baseline Characteristics and Demographics

	V116 + Placebo *N* = 386	PCV15 + PPSV23 *N* = 130	Total *N* = 516
Sex, *n* (%)			
Male	208 (53.9)	75 (57.7)	283 (54.8)
Age, y			
Mean (SD)	52.4 (9.6)	53.3 (8.9)	52.6 (9.4)
18–49, *n* (%)	119 (30.8)	41 (31.5)	160 (31.0)
50–64, *n* (%)	267 (69.2)	89 (68.5)	356 (69.0)
Race, *n* (%)			
White	294 (76.2)	101 (77.7)	395 (76.6)
Asian	57 (14.8)	17 (13.1)	74 (14.3)
Black or African American	16 (4.1)	2 (1.5)	18 (3.5)
Native Hawaiian or Other Pacific Islander	10 (2.6)	8 (6.2)	18 (3.5)
Other^a^	9 (2.3)	2 (1.5)	11 (2.1)
Ethnicity, *n* (%)			
Not Hispanic or Latino	280 (72.5)	93 (71.5)	373 (72.3)
Increased-risk conditions, *n* (%)			
1 increased-risk condition	326 (84.5)	108 (83.1)	434 (84.1)
Diabetes mellitus only	146 (37.8)	48 (36.9)	194 (37.6)
Chronic heart disease only	64 (16.6)	20 (15.4)	84 (16.3)
Chronic lung disease only	74 (19.2)	25 (19.2)	99 (19.2)
Chronic liver disease only	23 (6.0)	11 (8.5)	34 (6.6)
Chronic kidney disease only	19 (4.9)	4 (3.1)	23 (4.5)
≥2 increased-risk conditions	60 (15.5)	22 (16.9)	82 (15.9)

Abbreviations: PCV15, 15-valent pneumococcal conjugate vaccine; PPSV23, 23-valent pneumococcal polysaccharide vaccine; SD, standard deviation; V116, 21-valent adult-specific pneumococcal conjugate vaccine.

^a^Other includes American Indian or Alaska Native and those falling under multiple race categories.

In addition to increased-risk conditions, the proportions of participants with a diagnosis of alcoholism/alcohol use disorder in their medical history and/or who were current smokers were generally comparable between the V116 + placebo group (1.3% alcohol use disorder and 21.5% smoking) and the PCV15 + PPSV23 group (2.3% alcohol use disorder and 20.0% smoking). Reported medical history conditions among patients with ≥1 condition were also generally comparable between groups, with the most common conditions being diabetes mellitus (V116: 51.8%; PCV15 + PPSV23: 52.3%) and hypertension (V116: 48.7%; PCV15 + PPSV23: 49.2%).

### Immunogenicity

**Figure 3.**
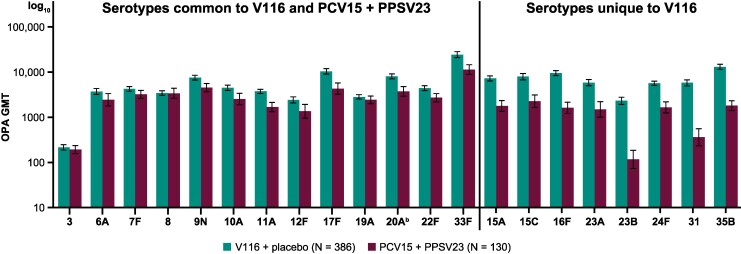
OPA GMTs at 30 d postvaccination for serotypes in V116.^a^ Error bars are within-group 95% CIs obtained by exponentiating the CIs of the mean of the natural log values based on the t-distribution. Abbreviations: CI, confidence interval; GMT, geometric mean titer; OPA, opsonophagocytic activity; PCV15, 15-valent pneumococcal conjugate vaccine; PPSV23, 23-valent pneumococcal polysaccharide vaccine; V116, 21-valent adult-specific pneumococcal conjugate vaccine. ^a^Postvaccination was at d 30 following V116 for the V116 + placebo group and at d 30 following PPSV23 (wk 12) for the PCV15 + PPSV23 group. ^b^Advances in serotyping methods led to further characterization of serotype 20 included in PPSV23 as 20A. This serotype is represented as 20 in PPSV23 labeling. Immune responses to serotype 20A were assessed.

**Figure 4.**
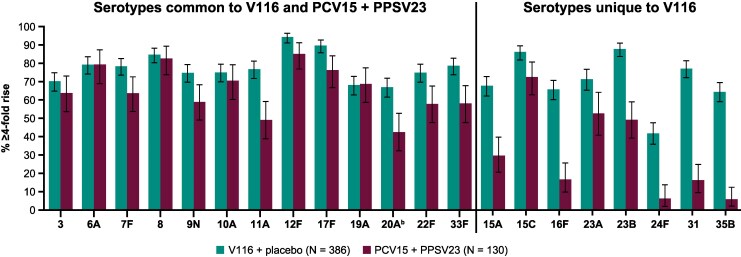
Proportion of participants with a ≥ 4-fold rise in OPA from baseline (d 1) to postvaccination.^a^ Error bars are within-group 95% CIs obtained by exponentiating the CIs of the mean of the natural log values based on the t-distribution. Abbreviations: CI, confidence interval; OPA, opsonophagocytic activity; PCV15, 15-valent pneumococcal conjugate vaccine; PPSV23, 23-valent pneumococcal polysaccharide vaccine; V116, 21-valent adult-specific pneumococcal conjugate vaccine. ^a^Postvaccination was at d 30 following V116 for the V116 + placebo group and at d 30 following PPSV23 (wk 12) for the PCV15 + PPSV23 group. ^b^Advances in serotyping methods led to further characterization of serotype 20 included in PPSV23 as 20A. This serotype is represented as 20 in PPSV23 labeling. Immune responses to serotype 20A were assessed.

V116 elicited immune responses at day 30 that were generally comparable to PCV15 + PPSV23 at week 12 for the 13 common serotypes, and numerically higher for the eight serotypes unique to V116, as measured by serotype-specific OPA GMTs (Figure 3; Supplementary Table 3). In addition, assessment of the proportion of participants with a ≥ 4-fold rise in serotype-specific OPA GMTs at 30 days postvaccination indicated that V116 was immunogenic for all 21 serotypes (Figure 4; Supplementary Table 3). This proportion for the V116 + placebo group was generally comparable to that observed for the PCV15 + PPSV23 group for the 13 common serotypes and was numerically higher than the PCV15 + PPSV23 group for the eight serotypes unique to V116.

**Figure 5.**
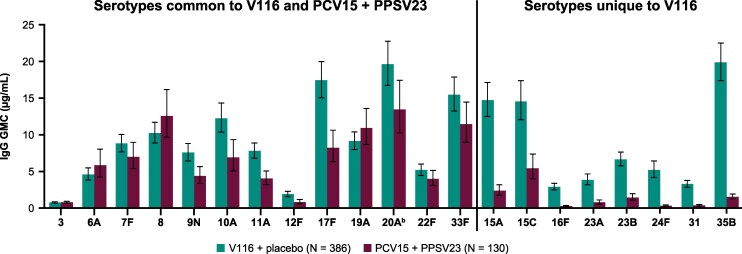
IgG GMCs at 30 d postvaccination for serotypes in V116.^a^ Error bars are within-group 95% CIs obtained by exponentiating the CIs of the mean of the natural log values based on the t-distribution. Abbreviations: CI, confidence interval; GMC, geometric mean concentration; IgG, immunoglobulin G; PCV15, 15-valent pneumococcal conjugate vaccine; PPSV23, 23-valent pneumococcal polysaccharide vaccine; V116, 21-valent adult-specific pneumococcal conjugate vaccine. ^a^Postvaccination was at d 30 following V116 for the V116 + placebo group and at d 30 following PPSV23 (wk 12) for the PCV15 + PPSV23 group. ^b^Advances in serotyping methods led to further characterization of serotype 20 included in PPSV23 as 20A. This serotype is represented as 20 in PPSV23 labeling. Immune responses to serotype 20A were assessed.

**Figure 6.**
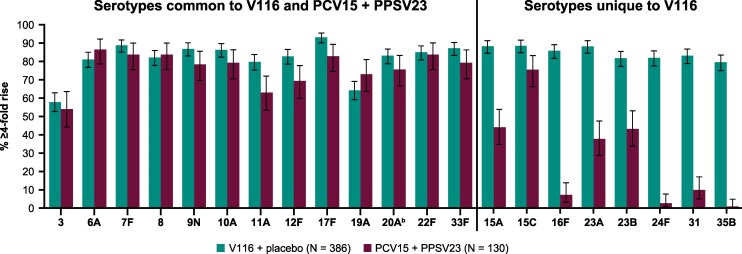
Proportion of participants with ≥4-fold rise in IgG GMCs from baseline (d 1) to postvaccination.^a^ Abbreviations: GMC, geometric mean concentration; IgG, immunoglobulin G; PCV15, 15-valent pneumococcal conjugate vaccine; PPSV23, 23-valent pneumococcal polysaccharide vaccine; V116, 21-valent adult-specific pneumococcal conjugate vaccine. ^a^Postvaccination was at d 30 following V116 for the V116 + placebo group and at d 30 following PPSV23 (wk 12) for the PCV15 + PPSV23 group. ^b^Advances in serotyping methods led to further characterization of serotype 20 included in PPSV23 as 20A. This serotype is represented as 20 in PPSV23 labeling. Immune responses to serotype 20A were assessed.

Serotype-specific IgG measurements 30 days postvaccination also demonstrated immunogenicity of V116 for all 21 serotypes contained in the vaccine, as assessed by GMCs (Figure 5; Supplementary Table 4) and the proportion of participants with a ≥ 4-fold rise in serotype-specific IgG from baseline (Figure 6; Supplementary Table 4). Both of these measures for the V116 + placebo group (measured at day 30 following V116) were generally comparable to those observed for PCV15 + PPSV23 (measured at day 30 following PPSV23 [week 12]) for the 13 common serotypes and were numerically higher for the eight serotypes unique to V116.

Subgroup analyses determined serotype-specific OPA GMTs for one and ≥2 concurrent increased-risk conditions for PD were generally consistent with the results observed in the overall population (Supplementary Tables 5 and 6). Similarly, OPA GMTs for each concurrent increased-risk condition were generally consistent with the overall population (data not shown).

### Safety

Most participants in both intervention groups reported at least one AE following any vaccination (Table 2). The proportions of participants with AEs, including injection-site AEs and systemic AEs, were numerically lower in the V116 + placebo group (68.7%) than in the PCV15 + PPSV23 group (90.8%). Vaccine-related AEs were also reported less frequently in participants who received V116 + placebo (62.4%) compared with those who received PCV15 + PPSV23 (86.2%). The proportion of participants in each intervention group who experienced SAEs did not exceed 5.4%. No SAE was considered by the investigator to be vaccine-related. There were no deaths reported for this study and no participants discontinued the study intervention due to an AE.

**Table 2.** Safety Summary

	V116 + Placebo		PCV15 + PPSV23	
	*n* (%)	95% CI^a^	*n* (%)	95% CI^a^
Participants in population	386	…	130	…
With one or more AE	265 (68.7)	63.8–73.3	118 (90.8)	84.4–95.1
Injection-site	206 (53.4)	…	107 (82.3)	…
Systemic	199 (51.6)	…	89 (68.5)	…
With no AE	121 (31.3)	…	12 (9.2)	…
With vaccine-related^b^ AEs	241 (62.4)	57.4–67.3	112 (86.2)	79.0–91.6
Injection-site	206 (53.4)	…	107 (82.3)	…
Systemic	154 (39.9)	…	70 (53.8)	…
With SAEs	10 (2.6)	1.2–4.7	7 (5.4)	2.2–10.8
With serious vaccine-related AEs	0 (0.0)	…	0 (0.0)	…
Who died	0 (0.0)	…	0 (0.0)	…
Discontinued vaccine due to an AE	0 (0.0)	…	0 (0.0)	…
Discontinued vaccine due to a vaccine-related AE	0 (0.0)	…	0 (0.0)	…
Discontinued vaccine due to a SAE	0 (0.0)	…	0 (0.0)	…
Discontinued vaccine due to a serious vaccine-related AE	0 (0.0)	…	0 (0.0)	…

Abbreviations: AE, adverse event; CI, confidence interval; PCV15, 15-valent pneumococcal conjugate vaccine; PPSV23, 23-valent pneumococcal polysaccharide vaccine; SAE, serious adverse event; V116, 21-valent adult-specific pneumococcal conjugate vaccine.

^a^Estimated CIs are calculated based on the exact binomial method proposed by Clopper and Pearson.

^b^Determined by the investigator to be related to the vaccine. All injection-site AEs and pyrexia solicited from d 1 to d 5 were considered vaccine-related.

**Figure 7.**
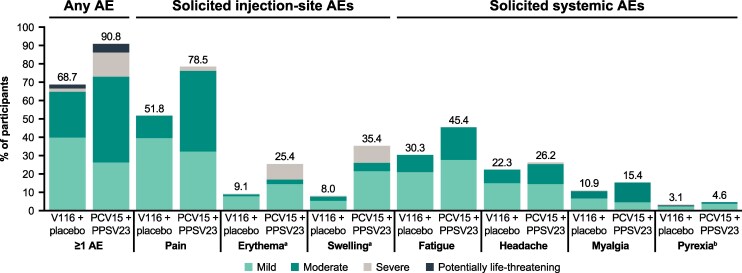
AEs by severity following any vaccination. V116 + placebo (*N* = 386); PCV15 + PPSV23 (*N* = 130); safety population. AEs include nonserious AEs occurring within 30 d of vaccination and SAEs occurring from d 1 until end of study participation. Solicited injection-site AEs and solicited systemic AEs were collected from d 1 to d 5 postvaccination. Severity described includes mild (Grade 1), moderate (Grade 2), severe (Grade 3), and potentially life-threatening (Grade 4). Abbreviations: AE, adverse event; PCV15, 15-valent pneumococcal conjugate vaccine; PPSV23, 23-valent pneumococcal polysaccharide vaccine; SAE, serious adverse event; V116, 21-valent adult-specific pneumococcal conjugate vaccine. ^a^Erythema and swelling were graded according to size and presented as intensity grade as follows: mild (0 to ≤5.0 cm); moderate (>5.0 to ≤10.0 cm); and severe (>10.0 cm). ^b^Pyrexia was defined as maximum temperature ≥100.4 °F (38.0°C), with ≥104.0 °F (40.0°C) defined as potentially life-threatening pyrexia.

Of the participants with solicited AEs, the majority experienced AEs of mild or moderate intensity in both the V116 + placebo group and the PCV15 + PPSV23 group (Figure 7). Overall, the proportions of participants with solicited AEs with a maximum intensity grade of severe were numerically lower in the V116 + placebo group than in the PCV15 + PPSV23 group. Solicited AEs by severity after each vaccination are shown in Supplementary Table 7. The majority of solicited AEs experienced following any vaccination were of short duration (≤3 days) in both intervention groups (Supplementary Figure 1). In general, participants in both intervention groups seemed to experience solicited AEs with similar maximum durations.

## DISCUSSION

This phase 3, randomized, international, active comparator-controlled trial identified that V116 was immunogenic and well tolerated in pneumococcal vaccine-naïve adults 18–64 years of age at increased risk of PD. V116 induced generally comparable immune responses to PCV15 + PPSV23 for the 13 serotypes common to both vaccine groups, and numerically higher responses for the eight serotypes unique to V116, based on OPA GMT and IgG GMC measurements. In general, immunogenicity findings showed similar trends to those reported for V116 in the general adult population, as well as for other PCVs in adults with underlying conditions [24–28].

V116 was well tolerated in adults with ≥1 increased-risk conditions. This is generally consistent with the safety outcomes observed for V116 in other studies [29, 30]. A numerically lower frequency of AEs, including systemic and injection-site AEs, was observed following vaccination with V116 + placebo compared with PCV15 + PPSV23. This finding is consistent with previous findings that reported a higher frequency of injection-site AEs with shorter intervals between pneumococcal vaccine doses [31, 32]. These immunogenicity and safety findings further support the use of V116 in people at increased risk of PD.

While previous recommendations for pneumococcal vaccination only included healthy adults ≥65 years of age and at-risk adults ≥19 years of age, recent updates to the US Advisory Committee on Immunization Practices guidelines recommend V116 vaccination for adults ≥50 years of age, in addition to all adults at increased risk of PD [33]. With this increase in the number of adults eligible for vaccination, V116 may help address healthcare disparities in adults, such as different rates of PD-related hospitalization and death due to the differences in the prevalence of underlying risk conditions across different communities and populations. Considering the high prevalence of underlying increased-risk conditions in the adult population [34, 35] that potentially remain undiagnosed [36, 37], working toward higher rates of adult coverage, potentially achieved with broader access to V116, may improve clinical outcomes in this population.

Statistical analyses were descriptive by design and, as such, the interpretability of between-group comparisons is limited. The majority of participants were White and not of Latino or Hispanic ethnicity. While this is representative of the racial and ethnic distribution of the participating countries, this limitation may affect the generalizability of the findings in certain regions. In addition, the study did not include individuals with human immunodeficiency virus (HIV) or with end-stage disease; however, evidence from studies administering V116 to adults with HIV and/or evaluating response to PCV15 in allogenic hematopoietic cell transplantation recipients suggests that V116 will likely be well tolerated and immunogenic in high-risk populations [38, 39]. This study did not evaluate immunogenicity between groups for the serotypes unique to the PCV15 + PPSV23 comparator group. Moreover, effectiveness data from real-world studies are needed to determine whether the immunogenicity of V116 translates into effective serotype-specific protection.

Overall, the results of this study show that V116 elicits robust immune responses and is well tolerated in individuals 18–64 years of age at increased risk of PD. Data in this population are likely valuable to policymakers and clinicians and may help to inform implementation practices. The findings further support the use of V116, an adult-specific PCV with broad serotype coverage, to prevent and potentially mitigate the burden of PD in both healthy and at-risk adults.

## Supplementary Material

ciaf604_Supplementary_Data

## Supplementary Data

Supplementary materials are available at *Clinical Infectious Diseases* online. Consisting of data provided by the authors to benefit the reader, the posted materials are not copyedited and are the sole responsibility of the authors, so questions or comments should be addressed to the corresponding author.

## Notes

***Author Contributions.*** P. T. S., D. F., and A. F. R. performed conceptualization. M. W. developed methodology, software development, performed formal analysis, and undertook data curation. M. W., P. T. S., and A. F. R. conducted validation of the results and reproducibility. P. T. S. and A. F. R. conducted the investigation. P. T. S., D. F., and A. F. R. provided the resources. P. T. S. performed writing of the original draft. M. W., P. T. S., D. F., and A. F. R. completed writing review and editing. P. T. S. prepared visualization. P. T. S. and D. F. provided supervision. D. F. managed project administration.

***Acknowledgments.*** The authors thank the study participants and their families who agreed to participate in this study. A full list of study investigators can be found in Supplementary Table 1. Medical writing assistance was provided by Alexa D'Ambra, PhD, and Cindy Cheung, MBBS (MD), and editorial assistance was provided by Ian Norton, PhD, all of Scion (a division of Prime, London, UK). This assistance was funded by Merck Sharp & Dohme LLC, a subsidiary of Merck & Co., Inc., Rahway, NJ, USA.

***Data availability statement.*** Merck Sharp & Dohme LLC, a subsidiary of Merck & Co., Inc., Rahway, NJ, USA (MSD), is committed to providing qualified scientific researchers access to anonymized data and clinical study reports from the company's clinical trials for the purpose of conducting legitimate scientific research. MSD is also obligated to protect the rights and privacy of trial participants and, as such, has a procedure in place for evaluating and fulfilling requests for sharing company clinical trial data with qualified external scientific researchers. The MSD data sharing website (available at: https://externaldatasharing-msd.com/) outlines the process and requirements for submitting a data request. Applications will be promptly assessed for completeness and policy compliance. Feasible requests will be reviewed by a committee of MSD subject matter experts to assess the scientific validity of the request and the qualifications of the requestors. In line with data privacy legislation, submitters of approved requests must enter into a standard data-sharing agreement with MSD before data access is granted. Data will be made available for request after product approval in the United States and European Union or after product development is discontinued. There are circumstances that may prevent MSD from sharing requested data, including country- or region-specific regulations. If the request is declined, it will be communicated to the investigator. Access to genetic or exploratory biomarker data requires a detailed, hypothesis-driven statistical analysis plan that is collaboratively developed by the requestor and MSD subject matter experts; after approval of the statistical analysis plan and execution of a data-sharing agreement, MSD will either perform the proposed analyses and share the results with the requestor or will construct biomarker covariates and add them to a file with clinical data that is uploaded to an analysis portal so that the requestor can perform the proposed analyses.

***Financial support.*** This work was supported by Merck Sharp & Dohme LLC, a subsidiary of Merck & Co., Inc., Rahway, NJ, USA. Although the sponsors formally reviewed a penultimate draft, the opinions expressed are those of the authors and may not necessarily reflect those of the sponsors. All coauthors approved the final version of the manuscript.

## References

[1] Yu X, Wang H, Ma S, Chen W, Sun L, Zou Z. Estimating the global and regional burden of lower respiratory infections attributable to leading pathogens and the protective effectiveness of immunization programs. Int J Infect Dis 2024; 194:107268. 10.1016/j.ijid.2024.107268 39413960

[2] Shoar S, Musher DM. Etiology of community-acquired pneumonia in adults: a systematic review. Pneumonia (Nathan Qld) 2020; 12:11. 33024653 10.1186/s41479-020-00074-3 PMC7533148

[3] Chen H, Matsumoto H, Horita N, Hara Y, Kobayashi N, Kaneko T. Prognostic factors for mortality in invasive pneumococcal disease in adult: a system review and meta-analysis. Sci Rep 2021; 11:11865. 34088948 10.1038/s41598-021-91234-y PMC8178309

[4] Cheong D, Young Song J. Pneumococcal disease burden in high-risk older adults: exploring the impact of comorbidities, long-term care facilities, antibiotic resistance, and immunization policies through narrative literature review. Hum Vaccin Immunother 2024; 20:2429235. 39631047 10.1080/21645515.2024.2429235 PMC11622649

[5] Ochoa-Gondar O, Torras-Vives V, de Diego-Cabanes C, et al Incidence and risk factors of pneumococcal pneumonia in adults: a population-based study. BMC Pulm Med 2023; 23:200. 37291502 10.1186/s12890-023-02497-2 PMC10251659

[6] Weycker D, Farkouh RA, Strutton DR, Edelsberg J, Shea KM, Pelton SI. Rates and costs of invasive pneumococcal disease and pneumonia in persons with underlying medical conditions. BMC Health Serv Res 2016; 16:182. 27177430 10.1186/s12913-016-1432-4 PMC4867996

[7] Hanquet G, Krizova P, Dalby T, et al Serotype replacement after introduction of 10-valent and 13-valent pneumococcal conjugate vaccines in 10 countries, Europe. Emerg Infect Dis 2022; 28:137–8. 34932457 10.3201/eid2801.210734 PMC8714201

[8] Vadlamudi NK, Chen A, Marra F. Impact of the 13-valent pneumococcal conjugate vaccine among adults: a systematic review and meta-analysis. Clin Infect Dis 2019; 69:34–49. 30312379 10.1093/cid/ciy872

[9] Shabil M, Gaidhane S, Ballal S, et al Mortality reduction with 23-valent pneumococcal polysaccharide vaccine: a systematic review and meta-analysis. Pneumonia 2024; 16:30. 39719643 10.1186/s41479-024-00149-5 PMC11669219

[10] Government of Canada . Regulatory decision summary for CAPVAXIVE. Available at: https://dhpp.hpfb-dgpsa.ca/review-documents/resource/RDS1722532649097. Accessed 10 November 2025.

[11] United States Food and Drug Administration . CAPVAXIVE™ (Pneumococcal 21-valent Conjugate Vaccine) Injection, for intramuscular use, prescribing information. Available at: https://www.fda.gov/media/179426/download?attachment. Accessed 10 November 2025.

[12] Therapeutic Goods Administration . CAPVAXIVE pneumococcal 21-valent conjugate vaccine 0.5 mL solution for pre-filled syringe. Available at: https://www.ebs.tga.gov.au/servlet/xmlmillr6?dbid=ebs/PublicHTML/pdfStore.nsf&docid=429290&agid=%28PrintDetailsPublic%29&actionid=1. Accessed 10 November 2025.

[13] European Commission . Public Health—Union Register of medicinal products. Available at: https://www.ema.europa.eu/en/medicines/human/EPAR/capvaxive. Accessed 10 November 2025.

[14] Gierke R. Current Epidemiology of Pneumococcal Disease among Adults, United States ACIP 2024. Available at: https://stacks.cdc.gov/view/cdc/148683. Accessed 10 November 2025.

[15] Food and Drug Administration . VAXNEUVANCE™ (pneumococcal 15-valent conjugate vaccine) prescribing information. Available at: https://www.fda.gov/media/150819/download. Accessed 10 November 2025.

[16] European Medicines Agency . VAXNEUVANCE (pneumococcal polysaccharide conjugate vaccine, 15-valent, adsorbed). Available at: https://www.ema.europa.eu/documents/product-information/vaxneuvance-epar-product-information_en.pdf. Accessed 10 November 2025.

[17] Food and Drug Administration . PNEUMOVAX 23 Prescribing information. Available at: https://www.fda.gov/media/80547/download. Accessed 10 November 2025.

[18] European Medicines Agency . Pneumovax 23 solution for injection in pre-filled syringe. Available at: https://www.medicines.org.uk/emc/product/9692/smpc. Accessed 10 November 2025

[19] Food and Drug Administration . PREVNAR 20 (Pneumococcal 20-valent Conjugate Vaccine) Prescribing Information. Available at: https://www.fda.gov/media/149987/download. Accessed 10 November 2025.

[20] Kobayashi M, Leidner AJ, Gierke R, et al Expanded recommendations for use of pneumococcal conjugate vaccines among adults aged ≥50 years: recommendations of the Advisory Committee on Immunization practices—United States, 2024. MMWR Morb Mortal Wkly Rep 2025; 74:1–8. 39773952 10.15585/mmwr.mm7401a1 PMC11709131

[21] Center for Disease Control and Prevention (CDC) . Summary of risk-based pneumococcal vaccination recommendations. Available at: https://www.cdc.gov/pneumococcal/hcp/vaccine-recommendations/risk-indications.html. Accessed 10 November 2025.

[22] Nolan KM, Bonhomme ME, Schier CJ, Green T, Antonello JM, Murphy RD. Optimization and validation of a microcolony multiplexed opsonophagocytic killing assay for 15 pneumococcal serotypes. Bioanalysis 2020; 12:1003–20. 32686954 10.4155/bio-2020-0024

[23] Nolan KM, Zhang Y, Antonello JM, et al Enhanced antipneumococcal antibody electrochemiluminescence assay: validation and bridging to the WHO reference ELISA. Bioanalysis 2020; 12:1363–75. 32975436 10.4155/bio-2020-0023

[24] Platt H, Omole T, Cardona J, et al Safety, tolerability, and immunogenicity of a 21-valent pneumococcal conjugate vaccine, V116, in healthy adults: phase 1/2, randomised, double-blind, active comparator-controlled, multicentre, US-based trial. Lancet Infect Dis 2023; 23:233–46. 36116461 10.1016/S1473-3099(22)00526-6

[25] Scott P, Haranaka M, Choi JH, et al A phase 3 clinical study to evaluate the safety, tolerability, and immunogenicity of V116 in pneumococcal vaccine-experienced adults 50 years of age or older (STRIDE-6). Clin Infect Dis 2024; 79:1366–74. 39082735 10.1093/cid/ciae383 PMC11650886

[26] Haranaka M, Yono M, Kishino H, et al Safety, tolerability, and immunogenicity of a 21-valent pneumococcal conjugate vaccine, V116, in Japanese healthy adults: a phase I study. Hum Vaccin Immunother 2023; 19:2228162. 37389808 10.1080/21645515.2023.2228162 PMC10316726

[27] Hammitt LL, Quinn D, Janczewska E, et al Immunogenicity, safety, and tolerability of V114, a 15-valent pneumococcal conjugate vaccine, in immunocompetent adults aged 18–49 years with or without risk factors for pneumococcal disease: a randomized phase 3 trial (PNEU-DAY). Open Forum Infect Dis 2022; 9:ofab605. 35146039 10.1093/ofid/ofab605 PMC8826015

[28] Hammitt LL, Quinn D, Janczewska E, et al Phase 3 trial to evaluate the safety, tolerability, and immunogenicity of V114, a 15-valent pneumococcal conjugate vaccine, followed by 23-valent pneumococcal polysaccharide vaccine 6 months later, in at-risk adults 18–49 years of age (PNEU-DAY): a subgroup analysis by baseline risk factors. Hum Vaccin Immunother 2023; 19:2177066. 36864601 10.1080/21645515.2023.2177066 PMC10026908

[29] Platt HL, Bruno C, Buntinx E, et al Safety, tolerability, and immunogenicity of an adult pneumococcal conjugate vaccine, V116 (STRIDE-3): a randomised, double-blind, active comparator controlled, international phase 3 trial. Lancet Infect Dis 2024; 24:1141–50. 38964361 10.1016/S1473-3099(24)00344-X

[30] Scott P, Haranaka M, Choi JH, et al A phase 3 clinical study to evaluate the safety, tolerability, and immunogenicity of V116 in pneumococcal vaccine-experienced adults 50 years of age or older (STRIDE-6). Clin Infect Dis 2024; 79:1366–74. 39082735 10.1093/cid/ciae383 PMC11650886

[31] Buchwald UK, Andrews CP, Ervin J, et al Sequential administration of Prevnar 13 and PNEUMOVAX 23 in healthy participants 50 years of age and older. Hum Vaccin Immunother 2021; 17:2678–90. 34019468 10.1080/21645515.2021.1888621 PMC8475587

[32] Miernyk KM, Butler JC, Bulkow LR, et al Immunogenicity and reactogenicity of pneumococcal polysaccharide and conjugate vaccines in Alaska native adults 55–70 years of age. Clin Infect Dis 2009; 49:241–8. 19522655 10.1086/599824

[33] Advisory Committee on Immunization Practices . ACIP Recommendations. Available at: https://www.cdc.gov/acip/vaccine-recommendations/?CDC_AAref_Val. Accessed 10 November 2025.

[34] GBD 2019 Diseases and Injuries Collaborators . Global burden of 369 diseases and injuries in 204 countries and territories, 1990–2019: a systematic analysis for the global burden of disease study 2019. Lancet 2020; 396:1204–22. 33069326 10.1016/S0140-6736(20)30925-9 PMC7567026

[35] Ward BW, Schiller JS, Goodman RA. Multiple chronic conditions among US adults: a 2012 update. Prevent Chronic Dis 2014; 11:E62. 10.5888/pcd11.130389 PMC3992293 24742395

[36] Maccio U, Andreas Meier C, Reinehr M, et al Clinically undiagnosed diseases in autopsies: frequency and risk factors. Arch Pathol Lab Med 2025; 149:60–6. 38576236 10.5858/arpa.2023-0429-OA

[37] Sahadevan P, Kumar Kamal V, Shankara Bagepally B, Kumari D, Pal A. Prevalence and risk factors associated with undiagnosed diabetes in India: insights from NFHS-5 national survey. J Glob Health 2023; 13:04135. 38063336 10.7189/jogh.13.04135 PMC10704946

[38] Wilck M, Cornely OA, Cordonnier C, et al A phase 3, randomized, double-blind, comparator-controlled study to evaluate safety, tolerability, and immunogenicity of V114, a 15-valent pneumococcal conjugate vaccine, in allogeneic hematopoietic cell transplant recipients (PNEU-STEM). Clin Infect Dis 2023; 77:1102-10. 37338158 10.1093/cid/ciad349 PMC10573722

[39] Pathirana J, Ramgopal M, Martin C, et al Safety, tolerability, and immunogenicity of an adult-specific pneumococcal conjugate vaccine, V116, in people living with HIV (STRIDE-7): a two-part, parallel-group, randomised, active comparator-controlled, international, phase 3 trial. Lancet HIV 2025; 12:e679–e690. 40953572 10.1016/S2352-3018(25)00165-1

