# Supplementary material for: A Phase 3, Randomized Trial Investigating the Safety, Tolerability, and Immunogenicity of V116, an Adult-Specific Pneumococcal Conjugate Vaccine, in Pneumococcal Vaccine-Naïve Adults 18–64 Years of Age at Increased Risk of Pneumococcal Disease, STRIDE-8
Source: Clin Infect Dis. 2025 Nov 10;82(2):e217–26. doi: 10.1093/cid/ciaf604 (PMC13016758; doi:10.1093/cid/ciaf604)
Supplement: ciaf604_Supplementary_Data [file ciaf604_supplementary_data.pdf]

Supplementary Table 1. List of Investigators

Primary Investigator/Facility Address[a]	Facility Description	Country	Protocol # - Site #	Sub-investigators	IEC Name and Address[b]	# of Participants Randomized
Jose Francisco Cardona,M.D.<br>Indago Research & Health Center, Inc<br>3700 West 12 Ave.<br>Suite 300<br>Hialeah, FL 33012<br>USA	Non-Hospital Clinical Research Site	USA	V116-008-0002	Yisel Castro Someillan, APRN/Juan Carlos Perez Sanchez/Evelio H. Sosa, MD	Advarra<br>6100 Merriweather Drive<br>Suite 600<br>Columbia, MD 21044<br>USA	28
Sashi K. Makam,M.D.<br>Mid Hudson Medical Research<br>17 Oakwood Terrace<br>New Windsor, NY 12553<br>USA	Non-Hospital Clinical Research Site	USA	V116-008-0008	Johanna Pagan	Advarra<br>6100 Merriweather Drive<br>Suite 600<br>Columbia, MD 21044<br>USA	6
David J. Morin,M.D.<br>Holston Medical Group<br>105 West Stone Drive<br>Clinical Research<br>Kingsport, TN 37660<br>USA	Non-Hospital Clinical Practice	USA	V116-008-0010	Emily J. Hubbs/Curtis M. Jantzi	Advarra<br>6100 Merriweather Drive<br>Suite 600<br>Columbia, MD 21044<br>USA	5
Olayemi O. Osiyemi,M.D.<br>Triple O Research Institute, P.A<br>2580 Metrocentre Boulevard<br>Suite 4<br>West Palm Beach, FL 33407<br>USA	Non-Hospital Clinical Practice	USA	V116-008-0011	Christina Campbell/Stephanie Martinez/Jose Menajovsky-Chaves	Advarra<br>6100 Merriweather Drive<br>Suite 600<br>Columbia, MD 21044<br>USA	2
Michael Livingston<br>SKY Integrative Medical Center/SKYCRNG	Non-Hospital Clinical Practice	USA	V116-008-0012	Christina Farmer, NP/Alisha Wilkes, NP	Advarra<br>6100 Merriweather Drive<br>Suite 600	9

4910 Jonesboro Road<br>Suite 601<br>Union City, GA 30291<br>USA					Columbia, MD 21044<br>USA	
Justin Pham/Suzanne Swan<br>EmVenio Research<br>2530 Meridian Parkway<br>Suite 300<br>Durham, NC 27713<br>USA	Non-Hospital Clinical Research Site	USA	V116-008-0018	Bobbi Jo Foust/Linda A Glaser/Susan Hernandez/Tamara Petronzio/Justin Pham	Advarra<br>6100 Merriweather Drive<br>Suite 600<br>Columbia, MD 21044<br>USA	5
Anton Elmer Grasch<br>Central Washington Health Services<br>Association d/b/a Confluence Health<br>820 North Chelan Avenue<br>Research Department<br>Wenatchee, WA 98801<br>USA	Hospital or Medical Center	USA	V116-008-0019	Kendra Morgan	Advarra<br>6100 Merriweather Drive<br>Suite 600<br>Columbia, MD 21044<br>USA	8
Kenneth R Boren<br>Aventiv Research<br>215 South Power Road<br>Suite 207<br>Mesa, AZ 85206<br>USA	Non-Hospital Clinical Research Site	USA	V116-008-0022	Molly Bertel/Kenneth R Boren/Brittany Cleveland/Sandra Gaiser/Chelsea M Hughes/Cole Valletta	Advarra<br>6100 Merriweather Drive<br>Suite 600<br>Columbia, MD 21044<br>USA	1
Bertin Mallet<br>G A Research Associates<br>1789 Mountain Road<br>Suite 206<br>Moncton, NB E1G 1A7<br>Canada	Non-Hospital Clinical Research Site	Canada	V116-008-0100	Ronald Leonard Bourgeois/Jocelyn Cormier/Julien Dupuis	Advarra<br>300 - 372 Hollandview Trail<br>Aurora, ON L4G 0A5<br>Canada	10

Ethel. Bellavance<br>Diex Recherche Victoriavile Inc.<br>39 rue Laurier Est<br>Suite 7<br>Victoriaville, QC G6P 6P6<br>Canada	Non-Hospital Clinical Research Site	Canada	V116-008-0102	Eve Betit/Karine Fortier	Advarra<br>300 - 372 Hollandview Trail<br>Aurora, ON L4G 0A5<br>Canada	5
Guy Tellier,F.R.C.P.<br>Manna Research Mirabel<br>101-13714 du Cure-Labelle<br>Mirabel, QC J7J 2K8<br>Canada	Non-Hospital Clinical Research Site	Canada	V116-008-0105	Luc Laperriere	Advarra<br>300 - 372 Hollandview Trail<br>Aurora, ON L4G 0A5<br>Canada	9
Peter Dzongowski,M.D.<br>Milestone Research Inc.<br>295 Saskatoon Street<br>London, ON N5W 6A2<br>Canada	Non-Hospital Clinical Research Site	Canada	V116-008-0106	Leslie Sinclair	Advarra<br>300 - 372 Hollandview Trail<br>Aurora, ON L4G 0A5<br>Canada	7
Richard Tytus<br>Hamilton Medical Research Group<br>700 Main Street East<br>Hamilton, ON L8M 1K7<br>Canada	Non-Hospital Clinical Practice	Canada	V116-008-0107	Stephen Tytus	Advarra<br>300 - 372 Hollandview Trail<br>Aurora, ON L4G 0A5<br>Canada	15
Ying Tung Sia<br>Diex Recherche Trois-Rivieres<br>6500, Boulevard Gene-H.-Kruger<br>Bureau 1<br>Trois-Rivieres, QC G9A 4P3<br>Canada	Non-Hospital Clinical Research Site	Canada	V116-008-0110	Ramon Alejandro Gisbert/Jean-francois Naud	Advarra<br>372 Hollandview Trail<br>Suite 300<br>Aurora, ON L4G 0A5<br>Canada	5
Dominique Tessier, M.D.<br>Clinique de médecine Urbaine du Quartier Latin	Hospital or Medical Center	Canada	V116-008-0111	Jean Guy Baril	Advarra<br>372 Hollandview Trail<br>Suite 300<br>Aurora, ON L4G 0A5<br>Canada	1

Hidemi Kanou<br>Medical corporation Applied Bio-Pharmatech Kurume Clinical Pharmacology Clinic<br>Kurume University school of Medicine<br>Building B67 Asahi-machi<br>Kurume, Fukuoka 830-0011<br>Japan	Non-Hospital Clinical Research Site	Japan	V116-008-0200	Hiroyuki Suzuki/Shuichi Yatsuga	Joint Institutional Review Board<br>1-14 Minamikubo<br>Sanyokochi Bld.3F<br>Kochi-shi, Kochi 781-0087<br>Japan	14
Akira Kato<br>Shimonoseki Medical Center<br>3-3-8 kamishinchi-chou<br>Shimonoseki Medical Center<br>Shimonoseki, Yamaguchi 750-0061<br>Japan	Hospital or Medical Center	Japan	V116-008-0201	Yuji Hisamatsu/ Hiroshi Iwamoto/Takashi Matsuda/Tomomi Nakamura/Kaoru Noda/Kuniyoshi Tanaka/Tomoyuki Uchida/Satoyoshi Yamashita/ koji harada	Japan Community Health care Organization<br>Shimonoseki Medical Center<br>IRB<br>3-3-8<br>Japan Community Health care Organization<br>Shimonoseki Medical Center<br>Shimonoseki, Yamaguchi<br>750-0061<br>Japan	22
BeLong, Cho /<br>Seoul National University Hospital<br>101, Daehak-ro, Jongno-gu<br>Seoul, 03080<br>South Korea	Hospital or Medical Center	South Korea	V116-008-0300	Seo Eun Hwang/Ji Young Kim	Institutional Review Board of Seoul National<br>University Hospital<br>IRB office, Center for Human Research<br>Protection<br>101, Daehak-ro, Jongno-gu,<br>Seoul, 03080<br>South Korea	6
Yong Bum, Park /<br>Kangdong Sacred Heart Hospital<br>150, Seongan-ro, Gangdong-gu,<br>Seoul, 05355<br>South Korea	Hospital or Medical Center	South Korea	V116-008-0301	Ga Young Ban	Institutional Review Board of Kangdong Sacred<br>Heart Hospital<br>150, Seongan-ro,<br>Gangdong-gu	6

					Seoul, 05355<br>South Korea	
Kwang Ha, Yoo /<br>Konkuk University Medical Center<br>120-1, Neungdong-ro, Gwangjin-gu<br>Seoul, 05030<br>South Korea	Hospital or Medical Center	South Korea	V116-008-0302	Won Hyeok Choe/Young-Il Jo/Sung Hea Kim/Youlim Kim	Institutional Review Board of Konkuk University<br>Medical Center<br>6th floor, A dong,<br>120-1, Neungdong-ro,<br>Gwangjin-gu<br>Seoul, 05030<br>South Korea	11
Yong Il, Hwang /<br>Hallym University Sacred Heart Hospital<br>22, 170beon-gil, Gwanpyeong-ro, Dongan-gu,<br>Anyang-si, Gyeonggi-do, 14068<br>South Korea	Hospital or Medical Center	South Korea	V116-008-0303	Kwang Yong Choi/Seung Hun Jang/Hwan Il Kim/Joo-Hee Kim/Ji Young Park/Sung Hoon Park	Institutional Review Board of Hallym University<br>Sacred Heart Hospital<br>4th Floor, Fourth Annex Building,<br>14, 176beon-gil,<br>Gwanpyeong-ro, Dongan-gu<br>Anyang-si, Gyeonggi-do, 14066<br>South Korea	8
Yang-Hyun, Kim/Byoungduck, Han /<br>Korea University Anam Hospital<br>73, Goryeodae-ro, Seongbuk-gu,<br>Seoul, 02841<br>South Korea	Hospital or Medical Center	South Korea	V116-008-0305	Byoungduck Han	Institutional Review Board of Korea University<br>Anam Hospital<br>5th Floor, New Building,<br>73 Goryeodae-ro,<br>Seongbuk-gu,<br>Seoul, 02841<br>South Korea	4
Krispin M Hajkowicz/Michael D Nissen<br>Royal Brisbane and Women's Hospital<br>Butterfield Street<br>QASIS, RBWH	Hospital or Medical Center	Australia	V116-008-0400	Damon Eisen/Krispin M Hajkowicz/Michael John Lane/Michael D Nissen	Metro North Health HREC A<br>Butterfield Street<br>Block 7, Level 7<br>RBWH, Queensland 4029<br>Australia	10

Herston, Queensland 4029<br>Australia						
Mark Theo Bloch,MB BS<br>Holdsworth House Medical Practice<br>26 College Street<br>Level 3<br>Sydney, New South Wales 2000<br>Australia	Non-Hospital Clinical Practice	Australia	V116-008-0402	Timothy G Barnes/Katharine Jane Bessey/Robert Alexander Burton/Jacqueline Engelander/Andrew Gowers/Gary Lee/Dick Cheong Quan/Shiva Rayar	Bellberry Human Research Ethics Committee<br>123 Glen Osmond Road<br>Eastwood, South Australia 5063<br>Australia<br>Holdsworth House Medical Practice<br>26 College Street<br>Level 3<br>Sydney, New South Wales 2000<br>Australia	4
Michael John Williams<br>Pacific Clinical Research Network - Rotorua<br>1289 Haupapa Street<br>Rotorua, Bay of Plenty 3010<br>New Zealand	Non-Hospital Clinical Research Site	New Zealand	V116-008-0500	Rosamund Carey/Paul Graham Hamilton/Dean Tasker	Health and Disability Ethics Committees<br>133 Molesworth Street<br>Ministry of Health<br>Thorndon, Wellington 6011<br>New Zealand	12
Benjamin James Image<br>Pacific Clinical Research Network – Forte<br>132 Peterborough Street<br>Forte 2, Level 2<br>Christchurch, Canterbury 8013<br>New Zealand	Non-Hospital Clinical Research Site	New Zealand	V116-008-0501	Joanne Therese Finlay/Christopher Hazlewood	Health and Disability Ethics Committees<br>133 Molesworth Street<br>Ministry of Health<br>Thorndon, Wellington 6011<br>New Zealand	11
Dean Richard Quinn<br>P3 Research - Wellington<br>121 Adelaide Road<br>1st Floor<br>Wellington, Wellington 6021<br>New Zealand	Non-Hospital Clinical Research Site	New Zealand	V116-008-0503	Joanna Victoria Joseph	Health and Disability Ethics Committees<br>133 Molesworth Street<br>Ministry of Health<br>Thorndon, Wellington 6011<br>New Zealand	12

Nigel Leslie Gilchrist<br>CGM Research Trust<br>Level 1<br>40 Stewart Street<br>Christchurch, Canterbury 8011<br>New Zealand	Hospital or Medical Center	New Zealand	V116-008-0505	None	New Zealand Health & Disability Committee (HDEC)<br>133 Molesworth Street<br>Wellington, Wellington 6011<br>New Zealand	16
Jackie Marie Kamerbeek<br>P3 Research - Tauranga<br>71 Tenth Avenue<br>Suite 11, Promed House<br>Tauranga, Bay of Plenty 3110<br>New Zealand	Non-Hospital Clinical Research Site	New Zealand	V116-008-0507	Santi Parbhu Lala/Katrina Louise Sandford/Mathanki Vivekananda	Health and Disability Ethics Committees<br>133 Molesworth Street<br>Ministry of Health<br>Thorndon, Wellington 6011<br>New Zealand	15
Richard Strawson Stubbs/Sarah (Polly) Penelope Bradford<br>P3 Research - Lower Hutt<br>1 Market Grove<br>Lower Hutt, Wellington 5010<br>New Zealand	Non-Hospital Clinical Research Site	New Zealand	V116-008-0508	Cheryl Archer/Sarah (Polly) Penelope Bradford/Joanna Victoria Joseph/Richard Strawson Stubbs	Health and Disability Ethics Committees<br>133 Molesworth Street<br>Ministry of Health<br>Thorndon, Wellington 6011<br>New Zealand	13
Piotr Piasecki<br>IN VIVO<br>Kaszubska 17H<br>Bydgoszcz, Kujawsko-pomorskie 85-046<br>Poland	Hospital or Medical Center	Poland	V116-008-0601	Grzegorz Gradzewicz/Dominika Korbal-Piasecka	Office for Registration of Medicinal Products, Medical Devices and Biocidal Products<br>Al. Jerozolimskie 181C<br>Warsaw 02-222<br>Poland	32
Jacek Mariusz Rolinski<br>Centrum Medyczne Medyk<br>Szopena 1<br>35-055<br>Rzeszów, Podkarpackie 35-055<br>Poland	Non-Hospital Clinical Practice	Poland	V116-008-0602	Michal Mazur/Stanislaw Mazur/Bernadetta Sobocka	Office for Registration of Medicinal Products, Medical Devices and Biocidal Products<br>Al. Jerozolimskie 181C<br>Warsaw 02-222<br>Poland	10

Grzegorz Kania<br>Clinmedica Research Sp. z. o. o.<br>Ogrodowa 21/23<br>Medical Office Building<br>Skierniewice, Lodzkie 96-100<br>Poland	Non-Hospital Clinical Research Site	Poland	V116-008-0603	Joanna Marzena Dziuda/Joanna Kania/Maciej Kania/Piotr Rolczyk/Maja Sawicka	Office for Registration of Medicinal Products, Medical Devices and Biocidal Products<br>Al. Jerozolimskie 181C<br>Warsaw 02-222<br>Poland	49
Szymon Placzek<br>Centrum Medyczne Pratia Katowice<br>13 Dabrowki<br>Katowice, Slaskie 40-084<br>Poland	Hospital or Medical Center	Poland	V116-008-0604	Elzbieta Blach	Office for Registration of Medicinal Products, Medical Devices and Biocidal Products<br>Al. Jerozolimskie 181C<br>Warsaw 02-222<br>Poland	11
Piotr Wilczek<br>Clinical Medical Research<br>Al.Korfantego 138<br>Katowice, Slaskie 40-156<br>Poland	Non-Hospital Clinical Research Site	Poland	V116-008-0605	Joanna Curyllo/Marta Kaleta-Richter/Agnieszka Mrozek/Daria Pietras	Office for Registration of Medicinal Products, Medical Devices and Biocidal Products<br>Al. Jerozolimskie 181C<br>Warsaw 02-222<br>Poland	14
Joanna Zdulska<br>MICS Centrum Medyczne Torun<br>Batorego 18-22<br>Torun, Kujawsko-pomorskie 87-100<br>Poland	Non-Hospital Clinical Practice	Poland	V116-008-0606	Jolanta Augustynowicz-Koziell	Office for Registration of Medicinal Products, Medical Devices and Biocidal Products<br>Al. Jerozolimskie 181C<br>Warsaw 02-222<br>Poland	0
Magdalena Dudek<br>Centrum Medyczne Pratia Bydgoszcz-Centrum Medyczne Pratia Bydgoszcz<br>Lochowskiego 7A<br>Bydgoszcz, Kujawsko-pomorskie	Non-Hospital Clinical Research Site	Poland	V116-008-0607	Jacek Duleba/Michał Gąsiorowski/Lucyna Losinska	Office for Registration of Medicinal Products, Medical Devices and Biocidal Products<br>Al. Jerozolimskie 181C	19

85-796<br>Poland					Warsaw 02-222<br>Poland	
Manuel Munoz<br>Hospital Dr. Hernán Henríquez Aravena<br>Manuel Montt 115<br>Temuco, Araucania 4781151<br>Chile	Hospital or Medical Center	Chile	V116-008-1001	Carolina Eugenia Chahin	Comite Etico Cientifico del Servicio de Salud Araucania Sur<br>Andres Bello 636<br>Temuco, Araucania 4791301<br>Chile	16
Carlos M. Perez , M.D.<br>Universidad San Sebastian - Providencia<br>Lota 2465<br>Providencia<br>Santiago, Region Metropolitana 7500000<br>Chile	Hospital or Medical Center	Chile	V116-008-1003	Loreto Aguirre Patricio Astudillo<br>Loreto Perez	Comite Etico Cientifico CEIC<br>Avenida Ricardo Lyon 291Ñuñoa, Region Metropolitana7770086<br>Chile	17
Magdalena De Aguirre<br>Centro de Investigacion Clinicadela Universidad Catolica<br>Av. Portugal 61<br>NULL<br>Santiago, Region M. de Santiago 8330034<br>Chile	Hospital or Medical Center	Chile	V116-008-1004	Luis Antonio Diaz Piga/Patricio Downey/Luigi Gabrielli/Diego Garcia-Huidobro	Comité Ético Científico de Ciencias de la Salud<br>340 Avenida Libertador Bernardo O'Higgins<br>4to piso<br>Santiago, Region M. de Santiago 8331150<br>Chile	13
Juan Carlos Prieto<br>Universidad de Chile - Hospital Clínico Universidad de Chile-Cardiology<br>Doctor Carlos Lorca Tobar 999, Independencia, Chile<br>Santiago, Region M. de Santiago<br>Chile	Hospital or Medical Center	Chile	V116-008-1006	Hernan Prat/Juan Carlos Prieto	Comité Ético Científico Hospital Clínico Universidad de Chile<br>999 Doctor Carlos Lorca Tobar<br>Santiago, Region M. de Santiago 8380494<br>Chile	8

Maite Oyonarte<br>Centro de Investigaciones Medicas Respiratorias (CIMER)<br>736 Matilde Salamanca<br>oficina 502 Piso 5<br>Providencia, Region M. de Santiago 7500657<br>Chile	Hospital or Medical Center	Chile	V116-008-1008	Rosa Maria Feijoo/Patricia Fernandez/Vicente Patricio Loayza fernandez/María Lavín Morgan/Monica Paredes/Paulina Trujillo	Comite de Etica Cientifico Metropolitano Oriente<br>364 Avenida Salvador<br>Providencia, Region M. de Santiago 7500922<br>Chile	14
Sergio George<br>CESFAM Esmeralda<br>Alpatacal 540<br>Colina, Santiago, Region M. de Santiago<br>Chile	Hospital or Medical Center	Chile	V116-008-1009	Sergio George/Enrique Vera	CEISH FACULTAD DE MEDICINA UNIVERSIDAD DE CHILE<br>1027 Avenida Independencia<br>COMITE DE ETICA<br>SANTIAGO, Region M. de Santiago 8380000<br>Chile	24
Absalon Rafael Silva Orellana, M.D.<br>Centro de Investigación del Maule-Centro de Investigación 2<br>30 Oriente edificio las rastras 1562<br>701,712,713,714,715<br>Talca, Maule 3481349<br>Chile	Hospital or Medical Center	Chile	V116-008-1010	Angel Oscar Gomez Cedre/Carolina Gonzalez/Giannina Mendoza	Comité Ético Científico CEIC<br>Av. Ricardo Lyon 2911, Ñuñoa, Santiago.<br>Santiago, Region M. de Santiago 7770086<br>Chile	11

[a]If the original investigator has been replaced, that investigator’s name is followed by a slash (/) and the replacement investigator’s name.

[b]Name(s) of Committee Chair/Alternate and Individual IEC members available upon request.

Supplementary Table 2. Inclusion and Exclusion Criteria for Relevant Risk Conditions

Relevant Increased-Risk Condition	Inclusion Criteria
Diabetes mellitus	Receiving treatment with ≥1 approved antidiabetic medication, with all HbA_{1c} measurements ≤9% within 6 months before first study vaccination (day 1).
Compensated chronic liver disease	Must meet ≥1 of the following:<br>Laboratory-confirmed FIB-4 score of ≥1.45 within 6 months before day 1[a].<br>Laboratory-confirmed APRI of >0.5 within 6 months before day 1[b].<br>Prior FibroTest® (FibroSure®) with Fibrosis Score >0.25 performed within 5 years before day 1.<br>Prior imaging (including FibroScan® with interpretable score >7 kPa) showing cirrhosis and/or fibrosis within 5 years before day 1.<br>Prior biopsy showing cirrhosis and/or fibrosis within 5 years before day 1.
Chronic obstructive pulmonary disease	Managed per local guidelines, and confirmed based on ≥1 of the following:<br>Spirometric data in the 5 years before day 1 showing post-bronchodilator FEV1/FVC ratio <0.7 and FEV1 ≥30% predicted, corresponding to spirometric Global Initiative for Chronic Obstructive Lung Disease Stage 1 to 3 by severity assessment [40].<br>A post-bronchodilator improvement in PEFR by <20% performed in the 5 years before day 1 based on WHO PEN Disease Interventions for Primary Health Care (2020) [41].
Chronic heart disease	Confirmed diagnosis managed per local guidelines including:<br>Cardiomyopathies documented within 5 years before day 1.<br>Congestive heart failure documented within 5 years before day 1.<br>Non-cyanotic congenital heart disease.
Chronic kidney disease	Confirmed diagnosis (>3 months duration) in 1 of the eligible KDIGO-recommended stages and GFR and albuminuria categories specified in table below within 6 months before day 1.

<table>
<tr><td colspan="2"></td><td colspan="3">Albuminuria</td></tr>
<tr><td colspan="2"></td><td>A1</td><td>A2</td><td>A3</td></tr>
<tr><td colspan="2">GFR (mL/min/1.73 m^2)</td><td><3 mg/mmol or <30 mg/g</td><td>3 to 30 mg/mmol or 30 to 300 mg/g</td><td>>30 mg/mmol or >300 mg/g</td></tr>
<tr><td>G1</td><td>≥90</td><td>Not eligible</td><td rowspan="3">Eligible</td><td rowspan="6">Not eligible</td></tr>
<tr><td>G2</td><td>60 to 89</td><td rowspan="2">Eligible</td></tr>
<tr><td>G3a</td><td>45 to 59</td></tr>
<tr><td>G3b</td><td>30 to 44</td><td rowspan="3">Not eligible</td><td rowspan="3">Not eligible</td></tr>
<tr><td>G4</td><td>15 to 29</td></tr>
<tr><td>G5</td><td><15</td></tr>
</table>

Mild or moderate persistent asthma	Managed per local guidelines and confirmed based on ≥1 of the following:<br>Documented reversible airflow obstruction on spirometry consistent with guidelines for the diagnosis of asthma performed within 5 years before first study vaccination (day 1).<br>A post-bronchodilator improvement in PEFR by ≥20% in the 5 years before first study vaccination (day 1), based on WHO PEN Disease Interventions for Primary Healthcare 2020 [41].

Medical Condition	Exclusion Criteria
Hepatitis	Elevation in pretreatment AST or ALT values >5 times ULN within 3 months before day 1.
Diabetic ketoacidosis	Within 3 months before day 1.
Hypoglycemia	≥2 episodes of severe, symptomatic hypoglycemia within 3 months before day 1.
Cardiac or cerebrovascular events	Myocardial infarction, acute coronary syndrome, transient ischemic attack, or ischemic or hemorrhagic stroke within 3 months before day 1.
Severe pulmonary hypertension	WHO functional class ≥3 or history of Eisenmenger syndrome.
Nephrotic disease	Including autoimmune-related kidney disease, chronic kidney failure, a reversible cause of kidney disease, nephrotic syndrome, or any ineligible KDIGO-recommended stages of GFR AND albuminuria categories specified in table below.

GFR (mL/min/1.73 m²)		Albuminuria A1 <3 mg/mmol or <30 mg/g	Albuminuria A2 3 to 30 mg/mmol or 30 to 300 mg/g	Albuminuria A3 >30 mg/mmol or >300 mg/g
G1	≥90	Not eligible	Eligible	Not eligible
G2	60 to 89	Eligible		
G3a	45 to 59			
G3b	30 to 44	Not eligible	Not eligible	
G4	15 to 29			
G5	<15			

Impaired immunological function	Including, but not limited to, congenital or acquired immunodeficiency, documented HIV infection, functional or anatomic asplenia, or autoimmune disease.
Coagulation disorder	Contraindicating intramuscular vaccination.
Malignancy	That is progressing or has required active treatment <3 years before randomization. Participants with basal cell and/or squamous cell carcinoma of the skin, or carcinoma in situ (including breast carcinoma and cervical cancer in situ) that have undergone potentially curative therapy are not excluded.

Abbreviations: ALT, alanine aminotransferase; APRI, AST-to-Platelet Ratio Index; AST, aspartate aminotransferase; FEV1, forced expiratory volume in 1 second; FIB-4, Fibrosis-4; FVC, forced vital capacity; GFR, glomerular filtration rate; HbA_{1c}, glycated hemoglobin; KDIGO, Kidney Disease Improving Global Outcomes; PEFR, peak expiratory flow rate; PEN: Package of Essential Noncommunicable; ULN, upper limit of normal; WHO, World Health Organization.

[a]FIB-4 score formula: age [years] × AST [IU/L]/platelet count [10^9/L] × $ALT^{1/2}$ [IU/L].

[b]APRI formula: AST/laboratory ULN for AST × 100/[platelet count/100].

Supplementary Table 3. Summary of OPA Responses at Day 1 and Day 30, Including GMFR and Percentage With ≥4-fold Rise in OPA

		V116 + Placebo (*N* = 386)			PCV15 + PPSV23 (*N* = 130)		
**Pneumococcal Serotype**	**Endpoint**	***n***	**Observed Response**	**95% CI**	***n***	**Observed Response**	**95% CI**
**13 Serotypes Common Between V116 and PCV15 + PPSV23**							
3	GMT (baseline)	361	16.9	15.0–19.0	125	20.5	16.5–25.5
	GMT (postvaccination)	352	216.2	188.6–247.8	104	192.9	156.7–237.4
	GMFR	334	8.3	7.2–9.5	102	6.1	4.8–7.7
	% ≥4-fold rise	334	70.1% (234/334)	64.8–74.9	102	63.7% (65/102)	53.6–73.0
6A	GMT (baseline)	329	127.5	102.6–158.4	114	116.5	82.3–164.9
	GMT (postvaccination)	347	3734.9	3204.3–4353.4	92	2443.8	1779.0–3357.1
	GMFR	297	22.8	18.6–28.0	82	17.1	11.9–24.7
	% ≥4-fold rise	297	79.1% (235/297)	74.1–83.6	82	79.3% (65/82)	68.9–87.4
7F	GMT (baseline)	365	167.6	135.5–207.4	124	245.7	170.5–354.2
	GMT (postvaccination)	355	4261.2	3781.6–4801.6	109	3218.5	2627.3–3942.6
	GMFR	341	20.4	16.9–24.6	107	10.5	7.5–14.7
	% ≥4-fold rise	341	78.3% (267/341)	73.5–82.6	107	63.6% (68/107)	53.7–72.6

8	GMT (baseline)	366	116.4	94.3–143.8	124	126.8	89.2–180.3
	GMT (postvaccination)	357	3460.8	3083.1–3884.7	106	3406.4	2635.8–4402.2
	GMFR	344	25.7	21.1–31.2	103	25.2	17.0–37.1
	% ≥4-fold rise	344	84.6% (291/344)	80.3–88.2	103	82.5% (85/103)	73.8–89.3
9N	GMT (baseline)	361	565.7	476.8–671.1	125	555.9	417.7–739.7
	GMT (postvaccination)	354	7553.9	6664.1–8562.5	107	4548.4	3696.8–5596.2
	GMFR	336	12.6	10.6–15.0	107	7.2	5.5–9.5
	% ≥4-fold rise	336	74.7% (251/336)	69.7–79.3	107	58.9% (63/107)	49.0–68.3
10A	GMT (baseline)	350	201.3	158.5–255.9	115	177.4	114.7–274.3
	GMT (postvaccination)	355	4502.0	3933.7–5152.4	107	2542.0	1897.0–3406.2
	GMFR	327	19.9	16.1–24.5	98	10.7	7.2–15.8
	% ≥4-fold rise	327	74.9% (245/327)	69.9–79.5	98	70.4% (69/98)	60.3–79.2
11A	GMT (baseline)	344	155.5	121.6–198.7	120	196.4	132.8–290.3
	GMT (postvaccination)	358	3761.7	3375.8–4191.7	106	1697.2	1338.4–2152.2
	GMFR	326	20.7	16.6–25.8	100	6.8	4.6–10.1
	% ≥4-fold rise	326	76.7% (250/326)	71.7–81.2	100	49.0% (49/100)	38.9–59.2
12F	GMT (baseline)	366	15.6	13.5–18.1	127	17.2	13.1–22.6
	GMT (postvaccination)	356	2432.3	2082.7–2840.6	107	1364.5	956.1–1947.4
	GMFR	343	95.0	79.9–113.0	107	48.6	32.5–72.6

	% ≥4-fold rise	343	94.2% (323/343)	91.1–96.4	107	85.0% (91/107)	76.9–91.2
17F	GMT (baseline)	350	353.5	293.3–426.1	124	240.7	171.4–338.1
	GMT (postvaccination)	354	10425.3	9099.2–11,944.7	104	4331.7	3248.3–5776.3
	GMFR	327	28.4	23.6–34.2	101	13.1	9.5–18.1
	% ≥4-fold rise	327	89.6% (293/327)	85.8–92.7	101	76.2% (77/101)	66.7–84.1
19A	GMT (baseline)	359	271.2	227.3–323.7	123	184.9	130.4–262.1
	GMT (postvaccination)	356	2837.2	2535.2–3175.2	106	2437.2	2008.5–2957.5
	GMFR	337	9.7	8.2–11.5	102	9.9	7.2–13.6
	% ≥4-fold rise	337	68.0% (229/337)	62.7–72.9	102	68.6% (70/102)	58.7–77.5
20A	GMT (baseline)	357	926.2	789.2–1087.0	125	953.3	723.5–1256.0
	GMT (postvaccination)	357	8091.5	7169.4–9132.3	99	3749.8	2932.4–4795.2
	GMFR	335	8.5	7.2–10.0	97	3.8	3.0–5.0
	% ≥4-fold rise	335	66.9% (224/335)	61.5–71.9	97	42.3% (41/97)	32.3–52.7
22F	GMT (baseline)	343	254.8	201.1–322.9	117	260.8	171.8–396.0
	GMT (postvaccination)	357	4432.7	3914.2–5020.0	108	2717.4	2201.7–3353.8
	GMFR	322	15.1	12.2–18.7	102	9.1	6.0–13.7
	% ≥4-fold rise	322	74.8% (241/322)	69.7–79.5	102	57.8% (59/102)	47.7–67.6
33F	GMT (baseline)	362	1994.8	1754.2–2268.4	126	1833.0	1447.4–2321.4
	GMT (postvaccination)	347	24,512.8	21,149.8–28,410.7	101	11,395.1	8884.5–14,615.3

	GMFR	330	12.3	10.4–14.5	100	5.5	4.1–7.2
	% ≥ 4-fold rise	330	78.5% (259/330)	73.7–82.8	100	58.0% (58/100)	47.7–67.8
**Eight Serotypes Unique to V116**							
15A	GMT (baseline)	336	686.4	577.2–816.3	113	768.6	578.7–1020.7
	GMT (postvaccination)	353	7274.6	6398.7–8270.4	104	1791.8	1361.7–2357.8
	GMFR	309	10.4	8.6–12.5	95	2.4	1.8–3.2
	% ≥4-fold rise	309	67.6% (209/309)	62.1–72.8	95	29.5% (28/95)	20.6–39.7
15C	GMT (baseline)	361	104.3	83.0–130.9	124	91.7	61.8–135.9
	GMT (postvaccination)	353	7923.1	6726.7–9332.3	107	2269.8	1651.8–3118.9
	GMFR	335	52.8	42.1–66.2	105	18.3	12.2–27.4
	% ≥4-fold rise	335	86.0% (288/335)	81.8–89.5	105	72.4% (76/105)	62.8–80.7
16F	GMT (baseline)	355	1010.6	863.5–1182.9	115	977.5	743.4–1285.4
	GMT (postvaccination)	356	9546.6	8396.2–10,854.6	105	1626.2	1224.4–2159.8
	GMFR	334	8.6	7.3–10.1	96	1.6	1.2–2.0
	% ≥4-fold rise	334	65.6% (219/334)	60.2–70.7	96	16.7% (16/96)	9.8–25.6
23A	GMT (baseline)	289	338.2	261.9–436.6	96	337.4	216.1–526.8
	GMT (postvaccination)	346	5875.3	5005.5–6896.2	100	1493.9	1009.1–2211.5
	GMFR	261	14.5	11.4–18.6	76	4.3	2.7–6.8
	% ≥4-fold rise	261	71.3% (186/261)	65.4–76.7	76	52.6% (40/76)	40.8–64.2

23B	GMT (baseline)	365	17.5	14.1–21.6	125	14.4	10.3–20.1
	GMT (postvaccination)	354	2316.9	1925.9–2787.3	108	117.0	73.4–186.4
	GMFR	341	83.6	66.4–105.3	106	6.2	4.3–9.0
	% ≥4-fold rise	341	87.7% (299/341)	83.7–91.0	106	49.1% (52/106)	39.2–59.0
24F	GMT (baseline)	314	1467.2	1250.6–1721.4	108	1647.4	1274.0–2130.2
	GMT (postvaccination)	354	5677.1	5098.2–6321.8	92	1666.8	1257.6–2209.1
	GMFR	293	4.0	3.4–4.7	81	0.9	0.7–1.2
	% ≥4-fold rise	293	41.6% (122/293)	35.9–47.5	81	6.2% (5/81)	2.0–13.8
31	GMT (baseline)	363	218.3	174.2–273.5	121	236.9	161.8–346.9
	GMT (postvaccination)	359	5803.9	4991.8–6748.1	105	360.7	233.0–558.5
	GMFR	344	23.2	18.4–29.1	99	1.5	1.2–2.0
	% ≥4-fold rise	344	77.0% (265/344)	72.2–81.4	99	16.2% (16/99)	9.5, 24.9
35B	GMT (baseline)	367	1886.6	1686.4–2110.6	125	1557.2	1248.7–1942.0
	GMT (postvaccination)	347	13,141.3	11,584.8–14,906.9	104	1812.1	1408.6–2331.2
	GMFR	337	7.2	6.3–8.3	102	1.2	1.0–1.4
	% ≥4-fold rise	337	64.4% (217/337)	59.0–69.5	102	5.9% (6/102)	2.2–12.4

Abbreviations: CI, confidence interval; GMFR, geometric mean fold rise; GMT, geometric mean titer; OPA, opsonophagocytic activity; PCV15, 15-valent pneumococcal conjugate vaccine; PPSV23, 23-valent pneumococcal polysaccharide vaccine; V116, 21-valent adult-specific pneumococcal conjugate vaccine.

Supplementary Table 4. Summary of IgG Responses at Day 1 and Day 30, Including GMFR and Percentage With ≥4-Fold Rise in IgG

		V116 + Placebo (*N* = 386)			PCV15 + PPSV23 (*N* = 130)		
**Pneumococcal Serotype**	**Endpoint**	***n***	**Observed Response**	**95% CI**	***n***	**Observed Response**	**95% CI**
**13 Serotypes Common Between V116 and PCV15 + PPSV23**							
3	GMC (baseline)	379	0.14	0.12–0.15	126	0.16	0.13–0.19
	GMC (postvaccination)	366	0.77	0.69–0.87	112	0.79	0.68–0.92
	GMFR	365	5.2	4.7–5.8	111	4.5	3.8–5.4
	% ≥4-fold rise	365	57.8% (211/365)	52.6–62.9	111	54.1% (60/111)	44.3–63.6
6A	GMC (baseline)	379	0.26	0.23–0.31	126	0.28	0.22–0.35
	GMC (postvaccination)	367	4.57	3.82–5.48	112	5.83	4.23–8.03
	GMFR	366	16.2	13.9–18.9	111	20.1	15.5–26.0
	% ≥4-fold rise	366	81.1% (297/366)	76.8–85.0	111	86.5% (96/111)	78.7–92.2
7F	GMC (baseline)	379	0.41	0.36–0.47	126	0.47	0.37–0.60
	GMC (postvaccination)	367	8.77	7.65–10.06	112	6.95	5.39–8.96
	GMFR	366	19.0	16.8–21.5	111	12.8	10.2–16.2
	% ≥4-fold rise	366	88.8% (325/366)	85.1–91.8	111	83.8% (93/111)	75.6–90.1

8	GMC (baseline)	379	0.68	0.60–0.78	126	0.80	0.64–1.00
	GMC (postvaccination)	367	10.20	8.89–11.71	112	12.50	9.68–16.14
	GMFR	366	14.8	12.9–17.0	111	15.5	11.5–21.0
	% ≥4-fold rise	366	82.2% (301/366)	77.9–86.0	111	83.8% (93/111)	75.6–90.1
9N	GMC (baseline)	379	0.38	0.33–0.44	126	0.44	0.34–0.56
	GMC (postvaccination)	367	7.53	6.42–8.83	112	4.35	3.35–5.66
	GMFR	366	19.2	16.8–22.0	111	9.9	7.9–12.5
	% ≥4-fold rise	366	86.9% (318/366)	83.0–90.2	111	78.4% (87/111)	69.6–85.6
10A	GMC (baseline)	379	0.58	0.51–0.67	126	0.59	0.47–0.74
	GMC (postvaccination)	367	12.18	10.36–14.32	112	6.88	5.07–9.34
	GMFR	366	20.1	17.6–23.0	111	11.0	8.6–13.9
	% ≥4-fold rise	366	86.3% (316/366)	82.4–89.7	111	79.3% (88/111)	70.5–86.4
11A	GMC (baseline)	379	0.67	0.59–0.76	126	0.74	0.61–0.92
	GMC (postvaccination)	367	7.78	6.80–8.89	112	4.04	3.22–5.07
	GMFR	366	11.7	10.3–13.3	111	5.6	4.5–7.0
	% ≥4-fold rise	366	79.8% (292/366)	75.3–83.8	111	63.1% (70/111)	53.4–72.0
12F	GMC (baseline)	379	0.09	0.08–0.10	126	0.08	0.07–0.11
	GMC (postvaccination)	367	1.90	1.58–2.28	112	0.84	0.60–1.18
	GMFR	366	17.7	15.3–20.5	111	8.1	6.2–10.6

	% ≥4-fold rise	366	82.8% (303/366)	78.5–86.5	111	69.4% (77/111)	59.9–77.8
17F	GMC (baseline)	379	0.57	0.50–0.65	126	0.56	0.45–0.70
	GMC (postvaccination)	367	17.35	15.06–19.98	112	8.20	6.32–10.64
	GMFR	366	29.6	25.9–33.8	111	13.9	10.9–17.6
	% ≥4-fold rise	366	93.2% (341/366)	90.1–95.5	111	82.9% (92/111)	74.6–89.4
19A	GMC (baseline)	379	1.28	1.16–1.41	126	1.29	1.07–1.56
	GMC (postvaccination)	367	9.11	7.98–10.41	112	10.88	8.70–13.60
	GMFR	366	7.1	6.3–8.0	111	8.1	6.5–10.0
	% ≥4-fold rise	366	64.2% (235/366)	59.1–69.1	111	73.0% (81/111)	63.7–81.0
20A	GMC (baseline)	379	1.28	1.14–1.44	126	1.52	1.25–1.86
	GMC (postvaccination)	367	19.51	16.73–22.75	112	13.38	10.27–17.43
	GMFR	366	15.1	13.2–17.2	111	8.7	7.0–10.8
	% ≥4-fold rise	366	83.1% (304/366)	78.8–86.8	111	75.7% (84/111)	66.6–83.3
22F	GMC (baseline)	379	0.23	0.20–0.26	126	0.25	0.19–0.32
	GMC (postvaccination)	367	5.17	4.44–6.02	112	3.98	3.08–5.14
	GMFR	366	19.7	17.0–22.8	111	13.2	10.2–17.3
	% ≥4-fold rise	366	85.0% (311/366)	80.9–88.5	111	83.8% (93/111)	75.6–90.1
33F	GMC (baseline)	379	1.00	0.87–1.14	126	1.02	0.82–1.26
	GMC (postvaccination)	367	15.38	13.23–17.88	112	11.40	8.99–14.46

	GMFR	366	14.7	13.0–16.6	111	11.0	8.8–13.8
	% ≥4-fold rise	366	87.2% (319/366)	83.3–90.4	111	79.3% (88/111)	70.5–86.4
**Eight Serotypes Unique to V116**							
15A	GMC (baseline)	379	0.61	0.53–0.69	126	0.58	0.46–0.74
	GMC (postvaccination)	367	14.65	12.52–17.13	112	2.37	1.77–3.18
	GMFR	366	23.6	20.6–27.1	111	3.7	3.0–4.6
	% ≥4-fold rise	366	88.3% (323/366)	84.5–91.4	111	44.1% (49/111)	34.7–53.9
15C	GMC (baseline)	379	0.56	0.48–0.66	125	0.57	0.43–0.75
	GMC (postvaccination)	367	14.48	12.07–17.38	112	5.42	3.98–7.37
	GMFR	366	24.2	20.8–28.2	110	9.2	7.3–11.7
	% ≥4-fold rise	366	88.5% (324/366)	84.8–91.6	110	75.5% (83/110)	66.3–83.2
16F	GMC (baseline)	379	0.16	0.14–0.19	126	0.17	0.14–0.21
	GMC (postvaccination)	367	2.90	2.48–3.38	111	0.27	0.21–0.35
	GMFR	366	16.0	14.1–18.1	110	1.6	1.4–1.9
	% ≥4-fold rise	366	85.8% (314/366)	81.8–89.2	110	7.3% (8/110)	3.2–13.8
23A	GMC (baseline)	379	0.17	0.15–0.20	126	0.20	0.15–0.25
	GMC (postvaccination)	367	3.85	3.18–4.66	112	0.79	0.56–1.11
	GMFR	366	20.9	18.2–24.0	111	3.8	2.9–4.9
	% ≥4-fold rise	366	88.3% (323/366)	84.5–91.4	111	37.8% (42/111)	28.8–47.5

23B	GMC (baseline)	379	0.37	0.32–0.42	126	0.37	0.29–0.46
	GMC (postvaccination)	367	6.62	5.75–7.63	112	1.46	1.09–1.96
	GMFR	366	16.6	14.3–19.2	111	3.7	3.0–4.6
	% ≥4-fold rise	366	81.7% (299/366)	77.3–85.5	111	43.2% (48/111)	33.9–53.0
24F	GMC (baseline)	379	0.28	0.24–0.32	126	0.30	0.23–0.38
	GMC (postvaccination)	367	5.18	4.17–6.43	112	0.34	0.26–0.43
	GMFR	366	18.0	15.5–20.9	111	1.1	1.1–1.2
	% ≥4-fold rise	366	82.0% (300/366)	77.6–85.8	111	2.7% (3/111)	0.6–7.7
31	GMC (baseline)	379	0.21	0.18–0.23	126	0.22	0.18–0.28
	GMC (postvaccination)	367	3.27	2.83–3.79	112	0.38	0.29–0.49
	GMFR	366	14.4	12.7–16.4	111	1.5	1.4–1.7
	% ≥ 4-fold rise	366	83.1% (304/366)	78.8–86.8	111	9.9% (11/111)	5.1–17.0
35B	GMC (baseline)	379	1.55	1.38–1.73	126	1.50	1.25–1.80
	GMC (postvaccination)	367	19.78	17.38–22.50	112	1.55	1.27–1.90
	GMFR	366	12.8	11.3–14.5	111	1.0	1.0–1.1
	% ≥4-fold rise	366	79.5% (291/366)	75.0–83.5	111	0.9% (1/111)	0.0–4.9

Abbreviations: CI, confidence interval; GMC, geometric mean concentration; GMFR, geometric mean fold rise; IgG, immunoglobulin G; PCV15, 15-valent pneumococcal conjugate vaccine; PPSV23, 23-valent pneumococcal polysaccharide vaccine; V116, 21-valent adult-specific pneumococcal conjugate vaccine.

Supplementary Table 5. OPA GMTs at 30 Days Postvaccination[a] for Participants With Single Increased-Risk Condition for Pneumococcal Disease[b]

	V116 + Placebo (*N* = 326)			PCV15 + PPSV23 (*N* = 108)		
**Pneumococcal Serotype**	***n***	**Observed GMT**	**95% CI[c]**	***n***	**Observed GMT**	**95% CI[c]**
**13 Serotypes Common Between V116 and PCV15 + PPSV23**						
3	299	220.2	190.2–254.9	88	181.0	143.8–227.9
6A	297	3974.4	3402.4–4642.5	78	2388.1	1706.9–3341.2
7F	303	4127.0	3616.0–4710.2	93	3295.4	2641.7–4110.8
8	304	3532.0	3118.7–4000.1	90	3716.6	2826.8–4886.4
9N	303	7725.1	6788.7–8790.7	91	4714.4	3717.6–5978.4
10A	303	4437.9	3807.9–5172.2	91	2660.6	1918.7–3689.5
11A	305	3919.8	3496.4–4394.5	91	1666.6	1272.1–2183.4
12F	305	2544.2	2160.8–2995.6	92	1494.6	1034.0–2160.2
17F	303	10,537.3	9099.7–12,202.0	88	4202.5	3052.2–5786.4
19A	304	2915.3	2594.2–3276.1	90	2519.7	2044.8–3105.0
20A	306	8013.9	7063.9–9091.7	83	3902.0	2950.7–5160.1
22F	304	4456.8	3887.9–5108.9	92	2757.8	2176.5–3494.5
33F	297	26,116.1	22,218.5–30,697.5	86	11,632.1	8808.4–15,360.9

**Eight Serotypes Unique to V116**						
15A	302	7352.0	6395.9–8451.1	89	1904.9	1405.8–2581.3
15C	302	7477.3	6286.6–8893.6	92	2271.2	1580.8–3263.3
16F	304	9470.3	8236.1–10,889.4	90	1601.5	1163.8–2203.9
23A	294	6070.3	5117.3–7200.6	85	1614.9	1058.5–2463.7
23B	301	2249.4	1844.2–2743.6	92	113.4	68.5–187.8
24F	303	5598.9	5002.7–6266.2	81	1820.2	1405.6–2357.1
31	306	5753.1	4906.6–6745.6	89	394.8	246.6–632.0
35B	297	13,393.3	11,682.8–15,354.2	88	1893.4	1429.3–2508.2

Abbreviations: CI, confidence interval; GMT, geometric mean titer; OPA, opsonophagocytic activity; PCV15, 15-valent pneumococcal conjugate vaccine; PPSV23, 23-valent pneumococcal polysaccharide vaccine; V116, 21-valent adult-specific pneumococcal conjugate vaccine.

[a]Postvaccination was at day 30 following V116 for the V116 + placebo group and at day 30 following PPSV23 (week 12) for the PCV15 + PPSV23 group.

[b]Increased-risk conditions refer to protocol-specific medical conditions required for eligibility (diabetes mellitus, chronic heart disease, chronic kidney disease, chronic liver disease, and chronic lung disease).

[c]The within-group 95% CIs were obtained by exponentiating the CIs of the mean of the natural log values based on the t-distribution.

Supplementary Table 6. OPA GMTs at 30 Days Postvaccination[a] for Participants with ≥2 Increased-Risk Conditions for Pneumococcal Disease[b]

	V116 + Placebo (*N* = 60)			PCV15 + PPSV23 (*N* = 22)		
**Pneumococcal Serotype**	***n***	**Observed GMT**	**95% CI[c]**	***n***	**Observed GMT**	**95% CI[c]**
**13 Serotypes Common Between V116 and PCV15 + PPSV23**						
3	53	195.1	133.2–285.7	16	273.3	168.0–444.7
6A	50	2582.1	1514.3–4402.7	14	2778.7	974.5–7923.4
7F	52	5134.6	3917.8–6729.4	16	2805.7	1591.0–4947.9
8	53	3078.9	2238.5–4234.7	16	2086.3	985.3–4417.7
9N	51	6612.3	4341.8–10,070.2	16	3709.6	2636.4–5219.7
10A	52	4894.1	3858.1–6208.4	16	1961.1	998.5–3851.8
11A	53	2968.2	2152.3–4093.5	15	1895.3	1243.6–2888.5
12F	51	1858.9	1153.9–2994.8	15	780.8	223.1–2732.9
17F	51	9784.1	6717.6–14,250.4	16	5116.1	2507.9–10,436.6
19A	52	2420.8	1677.7–3493.0	16	2021.2	1150.0–3552.4
20A	51	8573.3	5793.8–12,686.2	16	3050.5	1828.5–5089.2
22F	53	4297.4	3158.3–5847.4	16	2496.0	1576.7–3951.5
33F	50	16,824.9	11,848.0–23,892.3	15	10,126.8	5631.2–18,211.5

**Eight Serotypes Unique to V116**						
15A	51	6832.6	4865.0–9596.0	15	1246.0	644.9–2407.5
15C	51	11,163.9	6886.6–18,097.9	15	2260.8	1333.2–3834.0
16F	52	10,005.6	7141.2–14,018.8	15	1782.6	974.8–3260.1
23A	52	4885.2	3072.7–7766.6	15	960.9	301.4–3063.6
23B	53	2740.8	1636.4–4590.5	16	139.8	35.9–545.0
24F	51	6164.9	4380.9–8675.3	11	871.5	183.5–4138.9
31	53	6106.4	3858.4–9664.3	16	218.4	60.6–787.0
35B	50	11,739.2	8384.4–16,436.4	16	1423.5	788.2–2570.8

Abbreviations: CI, confidence interval; GMT, geometric mean titer; OPA, opsonophagocytic activity; PCV15, 15-valent pneumococcal conjugate vaccine; PPSV23, 23-valent pneumococcal polysaccharide vaccine; V116, 21-valent adult-specific pneumococcal conjugate vaccine.

[a]Postvaccination was at day 30 following V116 for the V116 + placebo group and at day 30 following PPSV23 (week 12) for the PCV15 + PPSV23 group.

[b]Increased-risk conditions refer to protocol-specific medical conditions required for eligibility (diabetes mellitus, chronic heart disease, chronic kidney disease, chronic liver disease, and chronic lung disease).

[c]The within-group 95% CIs were obtained by exponentiating the CIs of the mean of the natural log values based on the t-distribution.

Supplementary Table 7. Solicited Adverse Events by Severity After Each Vaccination

Category, *n* (%)	Intensity	V116 (*N* = 386)	Placebo (*N* = 374)	PCV15 (*N* = 130)	PPSV23 (*N* = 128)
>1 solicited AE	**Total**	**226 (58.5)**	**93 (24.9)**	**92 (70.8)**	**104 (81.3)**
	Mild	159 (41.2)	65 (17.4)	68 (52.3)	38 (29.7)
	Moderate	64 (16.6)	26 (7.0)	24 (18.5)	49 (38.3)
	Severe	3 (0.8)	1 (0.3)	0	17 (13.3)
	Potentially life-threatening	0	1 (0.3)	0	0
**Solicited Injection-Site AEs**					
Injection-site pain	**Total**	**191 (49.5)**	**31 (8.3)**	**82 (63.1)**	**93 (72.7)**
	Mild	150 (38.9)	24 (6.4)	67 (51.5)	35 (27.3)
	Moderate	41 (10.6)	7 (1.9)	15 (11.5)	55 (43.0)
	Severe	0	0	0	3 (2.3)
Injection-site erythema[a]	**Total**	**27 (7.0)**	**10 (2.7)**	**5 (3.8)**	**30 (23.4)**
	Mild	22 (5.7)	10 (2.7)	5 (3.8)	16 (12.5)
	Moderate	4 (1.0)	0	0	3 (2.3)
	Severe	1 (0.3)	0	0	11 (8.6)
Injection-site swelling[a]	**Total**	**25 (6.5)**	**10 (2.7)**	**10 (7.7)**	**45 (35.2)**
	Mild	16 (4.1)	9 (2.4)	7 (5.4)	28 (21.9)

	Moderate	8 (2.1)	1 (0.3)	3 (2.3)	5 (3.9)
	Severe	1 (0.3)	0	0	12 (9.4)
**Solicited Systemic AEs**					
Fatigue	**Total**	**95 (24.6)**	**54 (14.4)**	**32 (24.6)**	**42 (32.8)**
	Mild	69 (17.9)	37 (9.9)	24 (18.5)	22 (17.2)
	Moderate	26 (6.7)	17 (4.5)	8 (6.2)	20 (15.6)
	Severe	0	0	0	0
Headache	**Total**	**61 (15.8)**	**39 (10.4)**	**16 (12.3)**	**23 (18.0)**
	Mild	42 (10.9)	25 (6.7)	11 (8.5)	12 (9.4)
	Moderate	19 (4.9)	14 (3.7)	5 (3.8)	10 (7.8)
	Severe	0	0	0	1 (0.8)
Myalgia	**Total**	**31 (8.0)**	**17 (4.5)**	**7 (5.4)**	**15 (11.7)**
	Mild	19 (4.9)	11 (2.9)	5 (3.8)	2 (1.6)
	Moderate	11 (2.8)	6 (1.6)	2 (1.5)	13 (10.2)
	Severe	1 (0.3)	0	0	0
Pyrexia[b]	**Total**	**7 (1.8)**	**7 (1.9)**	**0**	**6 (4.7)**
	Mild	6 (1.6)	5 (1.3)	0	5 (3.9)
	Moderate	1 (0.3)	0	0	1 (0.8)
	Severe	0	1 (0.3)	0	0

Potentially life-threatening	0	1 (0.3)	0	0

Solicited injection-site AEs and solicited systemic AEs were collected from day 1 to day 5 postvaccination. Severity described includes mild (Grade 1), moderate (Grade 2), severe (Grade 3), and potentially life-threatening (Grade 4).

Abbreviations: AE, adverse event; PCV15, 15-valent pneumococcal conjugate vaccine; PPSV23, 23-valent pneumococcal polysaccharide vaccine; V116, 21-valent adult-specific pneumococcal conjugate vaccine.

[a]Erythema and swelling were graded according to size and presented as intensity grade as follows: mild (0 to ≤5.0 cm); moderate (>5.0 to ≤10.0 cm); and severe (>10.0 cm).

[b]Pyrexia was graded according to maximum temperature and presented as intensity grade as follows: mild, 38.0°C (100.4°F) to <38.5°C (101.3°F); moderate, 38.5°C (101.3°F) to <39.0°C (102.2°F); severe, 39.0°C (102.2°F) to <40.0°C (104.0°F); and potentially life-threatening, ≥40.0°C (104.0°F).

Supplementary Figure 1. AEs by maximum duration following any vaccination.

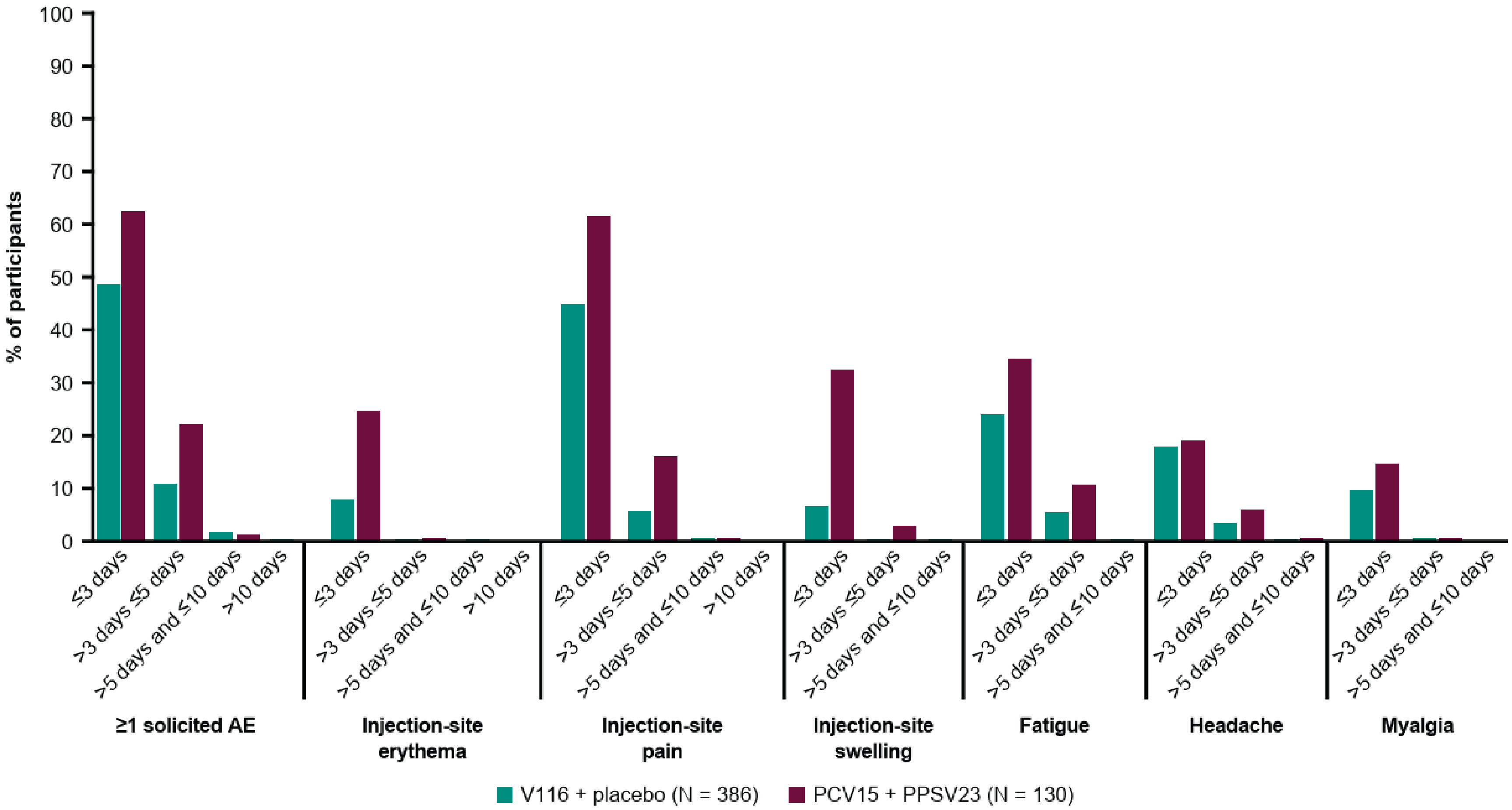

All-participants-as-treated population.
Abbreviations: AE, adverse event; PCV15, 15-valent pneumococcal conjugate vaccine; PPSV23, 23-valent pneumococcal polysaccharide vaccine; V116, 21-valent adult-specific pneumococcal conjugate vaccine.

Supplementary Methods 1.

Sample Size and Power for Safety Analyses

The sample size of approximately 375 participants in the 21-valent pneumococcal conjugate vaccine (V116) + placebo group and 125 participants in the 15-valent pneumococcal conjugate vaccine (PCV15) + 23-valent pneumococcal polysaccharide vaccine (PPSV23) group was selected to achieve a reasonably sized safety database in this population exposed to V116. The selected sample size allowed for the detection of a minimum level of serious adverse events (SAEs) with a high degree of confidence for the overall study. The probability of observing at least one SAE in this study was dependent on the number of participants vaccinated and the underlying incidence of participants with a SAE in the study population. Calculations assumed that 100% of the randomized participants will be evaluable for safety analyses: there is an 80% chance of observing at least one SAE among 375 participants in the V116 group if the underlying incidence of a SAE is 0.43% (one of 234 participants receiving the vaccine); there is a 50% chance of observing at least one SAE among 375 participants in the V116 group if the underlying incidence of a SAE is 0.18% (one of 542 participants receiving the vaccine). If no SAEs are observed among the 375 participants in the V116 group, this study would provide 97.5% confidence that the underlying percentage of participants with a SAE is <0.98% (one of 102 participants) in the V116 group.

Recruitment

Study recruitment was facilitated by including study investigators who had direct access to the patient population and/or had an established referral network with local specialists (such as hepatologists, endocrinologists, and cardiologists), who had

patients with the at-risk medical conditions described in the protocol. To facilitate recruitment, sites were provided study-specific informational materials for the participant (brochure, poster, flyer, dear participant letter, etc.).

Allocation Assignment and Blinding

The sponsor biostatistician generated the randomized allocation schedule, which included the allocation number ranges for study intervention assignment in a blocking factor of four. The study sites used a central electronic interactive response technology (IRT) system, which randomly assigned participants in a 3:1 ratio to receive either V116 on day 1 followed by placebo at week 8, or PCV15 on day 1 followed by PPSV23 at week 8. Randomization was stratified by participant’s age at the time of randomization (18–49 years of age or 50–64 years of age), and the type and number of increased-risk conditions for pneumococcal disease (diabetes mellitus only, chronic heart disease only, chronic kidney disease only, chronic liver disease only, chronic lung disease only, or ≥2 increased-risk conditions). The IRT system allocated participants to the appropriate vaccination group based on the next randomization number in the series within the group. A double-blind technique was used. Study vaccines were prepared and administered by unblinded qualified study site personnel who were not involved in subsequent participant assessments or study procedures. Investigators and the site staff who enrolled participants and performed clinical assessments, the data management team, and the participants remained blinded for the study duration.

Vaccines

V116 (Merck Sharp & Dohme LLC, a subsidiary of Merck & Co., Inc., Rahway, NJ, USA [MSD])) is a licensed 21-valent PCV containing 4 μg of purified capsular

pneumococcal polysaccharides (PnPs) from *Streptococcus pneumoniae* serotypes 3, 6A, 7F, 8, 9N, 10A, 11A, 12F, 15A, deOAc15B, 16F, 17F, 19A, 20A, 22F, 23A, 23B, 24F, 31, 33F, and 35B, all individually conjugated to cross-reacting material 197 (CRM_{197}) carrier protein [11].

PCV15 (MSD) is a 15-valent PCV that includes 2 µg of serotypes 1, 3, 4, 5, 6A, 7F, 9V, 14, 18C, 19A, 19F, 22F, 23F, and 33F, and 4 µg of serotype 6B, all individually conjugated to CRM_{197} carrier protein.

PPSV23 (MSD) is a 23-valent pneumococcal polysaccharide vaccine that contains 25 µg of PnPs from serotypes 1, 2, 3, 4, 5, 6B, 7F, 8, 9N, 9V, 10A, 11A, 12F, 14, 15B, 17F, 18C, 19A, 19F, 20, 22F, 23F, and 33F, and does not use a carrier protein [15, 17].

The serotype de-O-acetylated 15B polysaccharide in V116 has a similar molecular structure to serotype 15C, and, as such, antibodies to serotype 15C were measured to evaluate immune responses to de-O-acetylated 15B. Advances in serotyping methods led to further characterization of serotype 20 included in PPSV23 as serotype 20A; this was represented as serotype 20 in PPSV23 labeling and as serotype 20A in V116 labeling. Immune responses to serotype 20A were assessed in this study.

Interim Analyses and Stopping Guidelines

A periodic review of safety and tolerability data across the V116 phase 3 adult program was conducted by an independent, unblinded, external data monitoring committee (DMC). Unblinded immunogenicity data were made available to the DMC on request to enable a benefit–risk assessment. The DMC served as the primary

reviewer of the results of the ongoing safety reviews and could make recommendations for continuation of the study (with or without protocol modifications) or discontinuation of the study to an executive oversight committee of the sponsor.
